# Simulation of the Dynamics of Primary Immunodeficiencies in B Cells

**DOI:** 10.3389/fimmu.2018.01785

**Published:** 2018-08-02

**Authors:** Gabriel Ndipagbornchi Teku, Mauno Vihinen

**Affiliations:** Department of Experimental Medical Science, BMC B13, Lund University, Lund, Sweden

**Keywords:** primary immunodeficiency, systems analysis, models, biological, B-cell network model, semi-quantitative network simulation, B-cell network simulation

## Abstract

Primary immunodeficiencies (PIDs) are a group of over 300 hereditary, heterogeneous, and mainly rare disorders that affect the immune system. Various aspects of immune system and PID proteins and genes have been investigated and facilitate systems biological studies of effects of PIDs on B cell physiology and response. We reconstructed a B cell network model based on data for the core B cell receptor activation and response processes and performed semi-quantitative dynamic simulations for normal and B cell PID failure modes. The results for several knockout simulations correspond to previously reported molecular studies and reveal novel mechanisms for PIDs. The simulations for CD21, CD40, LYN, MS4A1, ORAI1, PLCG2, PTPRC, and STIM1 indicated profound changes to major transcription factor signaling and to the network. Significant effects were observed also in the BCL10, BLNK, BTK, loss-of-function CARD11, IKKB, MALT1, and NEMO, simulations whereas only minor effects were detected for PIDs that are caused by constitutively active proteins (PI3K, gain-of-function CARD11, KRAS, and NFKBIA). This study revealed the underlying dynamics of PID diseases, confirms previous observations, and identifies novel candidates for PID diagnostics and therapy.

## Introduction

The human immunome covers the entirety of genes and proteins that are essential for innate and adaptive immunity (1). Many of these proteins are involved in extensive interaction networks. We have previously defined and characterized the essential immunome interactome, i.e., the totality of interactions in the immune system (2). These data can be used for many purposes including studies of the dynamics of the immune response in health and disease. The immunome interactome is not stable; it varies between cell types and even within them depending on the timing and localization of expressed and active proteins.

B cells produce antibodies. The membrane-bound antibody component of the B cell receptor (BCR) recognizes foreign antigens and triggers a cascade of signal transduction events that lead to the activation as well as nuclear transport of specific transcription factors (TFs) (3). In the nucleus, these TFs activate transcription of B cell proliferation and response genes. B cells can be divided into subpopulations based on the expression of surface markers. B cell maturation is a complex process that proceeds from hematopoietic stem cells *via* pro- and pre-cell stages to immature and mature B cells and finally to plasma or memory cells. The B cell subtypes play different and sometimes overlapping roles in immune response by virtue of their differing master regulator TFs (4) and surface receptors (5–7).

Primary immunodeficiencies (PIDs) are intrinsic diseases of the immune system, mostly rare and typically with heterogeneous phenotypes. Already over 300 PIDs have been described. Disease-causing variants have been collected to IDbases (8) and some other databases. Differential diagnosis of PIDs is often difficult because of overlapping signs and symptoms, sometimes only a genetic test can provide the definitive answer. There are some classification schemes for PIDs, including the one from the International Union of Immunological Societies (IUIS) expert committee for PIDs (9) and the network-based classification that clusters the diseases based on signs, symptoms and laboratory parameters (10).

The reconstruction of cellular networks in systems biology facilitates simulations where diseases are modeled as perturbations or alterations (11, 12). Since experimental studies are very tedious, they have been limited to small networks. Mathematical network simulations can offer insight into the dynamics of biomolecular interactions in cellular processes both in health and disease. Some protein–protein interaction (PPI) networks in B cells and their role in various diseases have been studied (13–15). These interactions can be disrupted by disease-causing variations. Quantitative dynamics studies are computationally intensive when the number of parameters is extensive. Further problems occur because of lack of kinetic parameters and reaction constants. Therefore, other approaches have been developed to study larger networks of up to hundreds of nodes by using qualitative and semi-quantitative dynamics (16–18).

Here, we employed the semi-quantitative method of normalized HillCube Boolean approach (19) to simulate the dynamics during the activation of B cells. Variants that cause PIDs were used to study the effects of PID perturbations. We conducted synchronous update simulations and validated them *in silico*. The simulations reproduced known trends due to variations in PIDs in essential signal transduction pathways during B cell development from pre- to mature B cell (20). Moreover, we found several novel proteins affected by PIDs and detected novel PID candidates.

The content of this article is the full paper of part of a published doctoral thesis (21).

## Results

### The Primary BCR Network

**Figure 1:**
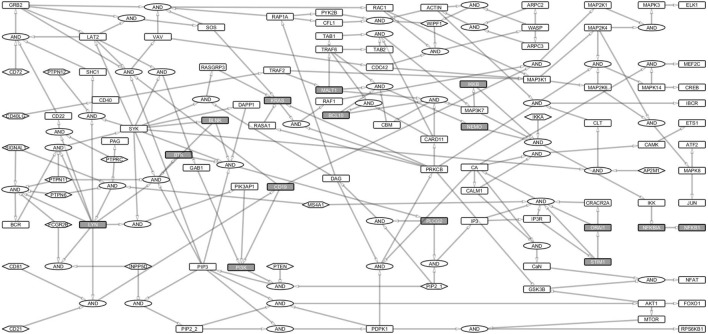
B cell activation Boolean network model. The network consists of 222 links and 144 nodes (including the AND Boolean operator), 17 of which are input nodes that have no links pointing to them. The Boolean network represents the B cell activation events. The rectangles for non-primary immunodeficiencies (PIDs) are white and for PID proteins are gray. Ellipses denote the AND gates. Input nodes are represented by hexagonal boxes. Activating links have pointed arrows while inhibiting links have blunt heads. Signal 1 represents B cell receptor (BCR) complex. Since the network focuses on BCR, its coreceptor (CD19/CD21/CD81), and costimulatory receptor, CD40 signaling events, only major events, e.g., for survival signaling and response have been fully considered.

To investigate B cell signal transduction, we started by building the central networks based on data from literature. Then we constructed reaction equations for the primary BCR activation and response and converted them into a Boolean network model (Table S1 in Supplementary Material). These interactions were manually defined as Boolean equations using the sum-of-product form similar to our previous study on T-cell networks (22). Proteins were treated as Boolean variables in the hypergraph. Activating (+) and inhibiting (−) interactions were represented as edges with pointed or blunt arrowheads, respectively. Each edge originates from a source and points to a target and indicates the direction of signal transduction (Figure 1). Multiple edges pointing at the same node represent summed interactions and were represented by the OR operator, while multiple incoming edges that together activate or inhibit a protein were connected to the target using the AND gate. Totally 17 input nodes in the network did not have incoming links (Figure 1).

We analyzed the structure of the network and the signaling paths between the initial events of the BCR activation and the late events of major TFs required for the expression of response genes. The BCR complex, its coreceptor, CD19/21/81, and the CD40 costimulatory receptor are involved in the initial events, while the TFs ELK1, AP1, NFAT, and NF-κB control the late events of B cell activation (23).

The BCR is activated when it binds to an antigen (signal 1). Another signal (signal 2) through the costimulatory receptor, *via* CD40, cytokines and CD19/CD21/CD81 complex is needed to elicit survival, and response (24, 25). The multiple paths from receptors to TFs guarantee a fail-safe and robust B cell activation (26–28). The sensitivity of gene activation is likely modulated by different routes (29).

**Figure 2:**
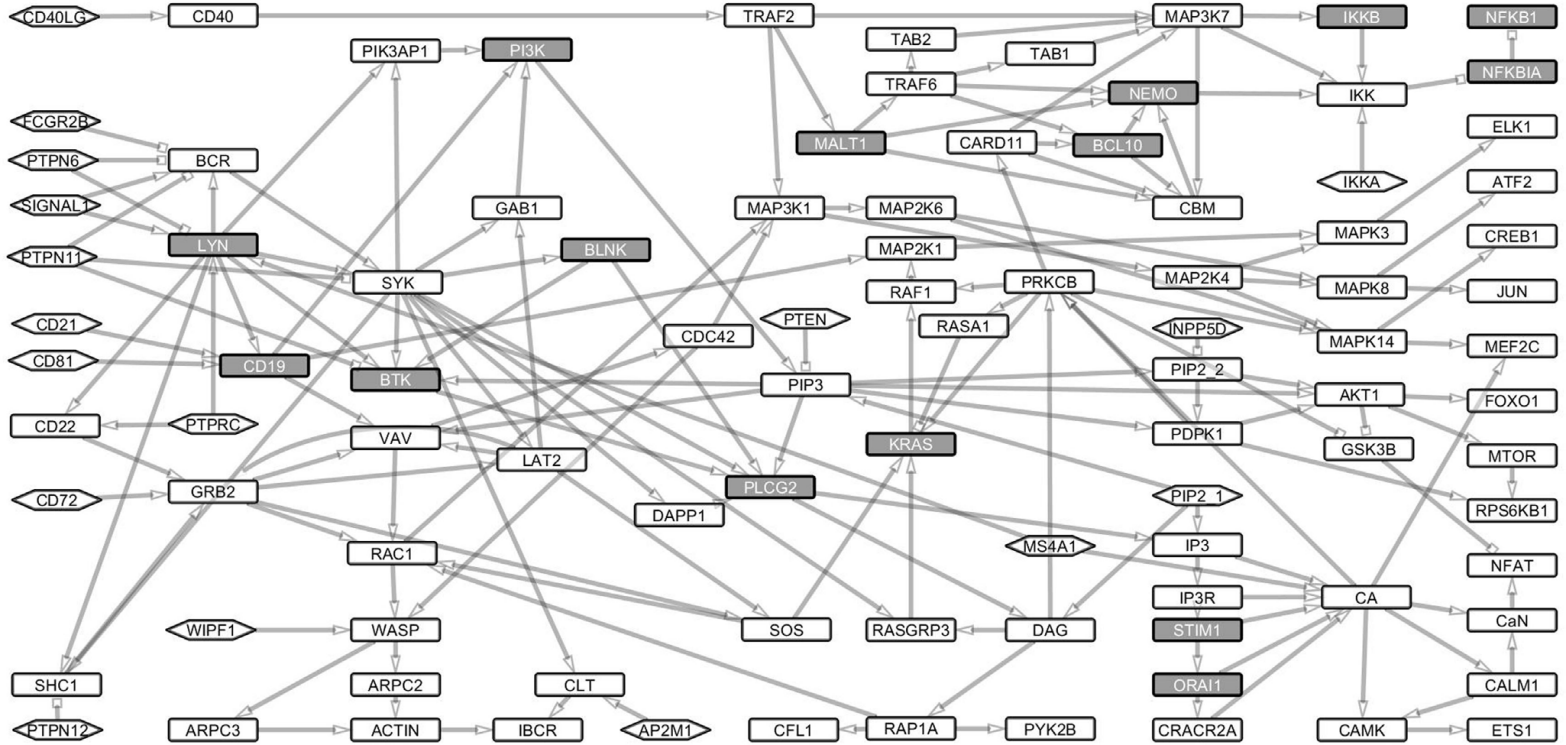
Boolean model transformed into its underlying interaction graph. The network consists of nodes and links derived from the Boolean network model without the AND operator. The interaction graph contains 107 nodes and 188 links and represents the underlying interaction network of the model. The nodes are as described in Figure 1. The network shows the paths through which signals from the receptors are channeled through the network to the transcription factors, which turn on the response genes.

We identified paths from signals 1 and 2 to the major response TFs including ATF2, CREB1, ELK1, ETS1, FOXO1, JUN, MEF2C, MTOR, NFKB1, and NFAT. For this purpose, we converted the Boolean network into an interaction network (Figure 2) to capture the dependencies, interactions, and thus, the paths through which signals are transduced. The entire interaction network is a single strongly connected component of 97 nodes interconnected by 168 links (Table S1 in Supplementary Material).

Signaling loops indicate the dynamic nature of a network. We analyzed proteins that are essential for transducing signals between the receptor components (BCR and CD40LG) to downstream TFs. Proteins whose Boolean update equations are along most of the loops were considered essential. In total, we found 6,542 such loops of which the longest spans 26 nodes and the shortest 2 nodes. The mean length of the loops is 15 nodes. All the loops included PID proteins (Table S2 in Supplementary Material). SYK, PLCG2, LYN, PI3, PI3K, IP3, and CA are the nodes that are most frequently identified along the loops. These results show that the known PID proteins are central for the B cell pathways and thus harmful variants in these proteins would be detrimental for the signal flow.

### *In Silico* Validation of Reconstructed Network and Identification of the Wild-Type Attractor

The engagement of the BCR, its coreceptor, CD19/CD21/CD81 complex and the CD40 stimulatory receptor, triggers a series of signal transduction cascades in B cells (3), which are captured in our reconstructed network (Figure 1; Table S1 in Supplementary Material). The signaling cascades activate numerous TFs, including ATF2 (30), CREB1 (31), ELK1 (31), ETS1 (32), FOXO1 (33), JUN (34, 35), MEF2C (36), NFKB1 (37), and NFAT (38). The reconstructed network is considered cogent when the major TFs and the signaling components that lead to their activation are turned on.

To ensure that our model reproduces B cell activation, we performed a large number of simulations by iteratively modifying the parameters and initial states of the input nodes, and ensuring that the network represented the main signaling events. We used normalized HillCube dynamic simulations (19) with signals 1 and 2 turned on and validated the simulations *in silico*. In addition, we performed simulations by turning signal 1 on and signal 2 off and *vice versa*. When signal 1 was turned off, ATF2 and JUN were turned on, while all other TFs were turned off, indicating the importance of the signal flow *via* this route. This effect is corroborated by previous experiments showing that when the CD40 receptor is stimulated by interaction with CD40LG the MAPK signal transduction pathways are activated. ATF2 and JUN are both activated by the MAPK signaling pathways (39). Although all other TFs were turned on when signal 2 was off, NFKB1, a very important survival signal for B cells, was turned off. This result is supported by experiments showing that the NF-κB pathway is activated by signal 1 (40). These simulations were accompanied by the tuning of the parameters such that the simulation results should comply with previously published experimental. We used the default values of the parameters for all equations except for those in Table 1.

**Table 1:** Tuned parameters of nodes in the Odefy-simulated B cell network model.

Influenced node	Influencing node(s)	τ	*n*	*k*
LYN	PTPRC	10	20	0.9
BCR	PTPN6	1	32	0.9
BCR	PTPN11	1	32	0.9
BCR	FCGR2B	1	1	0.9
PIP2_2	INPP5D	16	32	0.9

*LYN, LYN proto-oncogene, Src-family tyrosine kinase; PTPRC, protein tyrosine phosphatase, receptor type C; PTPN6, protein tyrosine phosphatase, non-receptor type 6; PTPN11, protein tyrosine phosphatase, non-receptor type 11; PIP2_2, PIP_2, phosphatidylinositol (3,4)-bisphosphate; FCGR2B, Fc fragment of IgG receptor IIb; INPP5D, inositol polyphosphate-5-phosphatase D; BCR, B cell receptor*.

**Figure 3:**
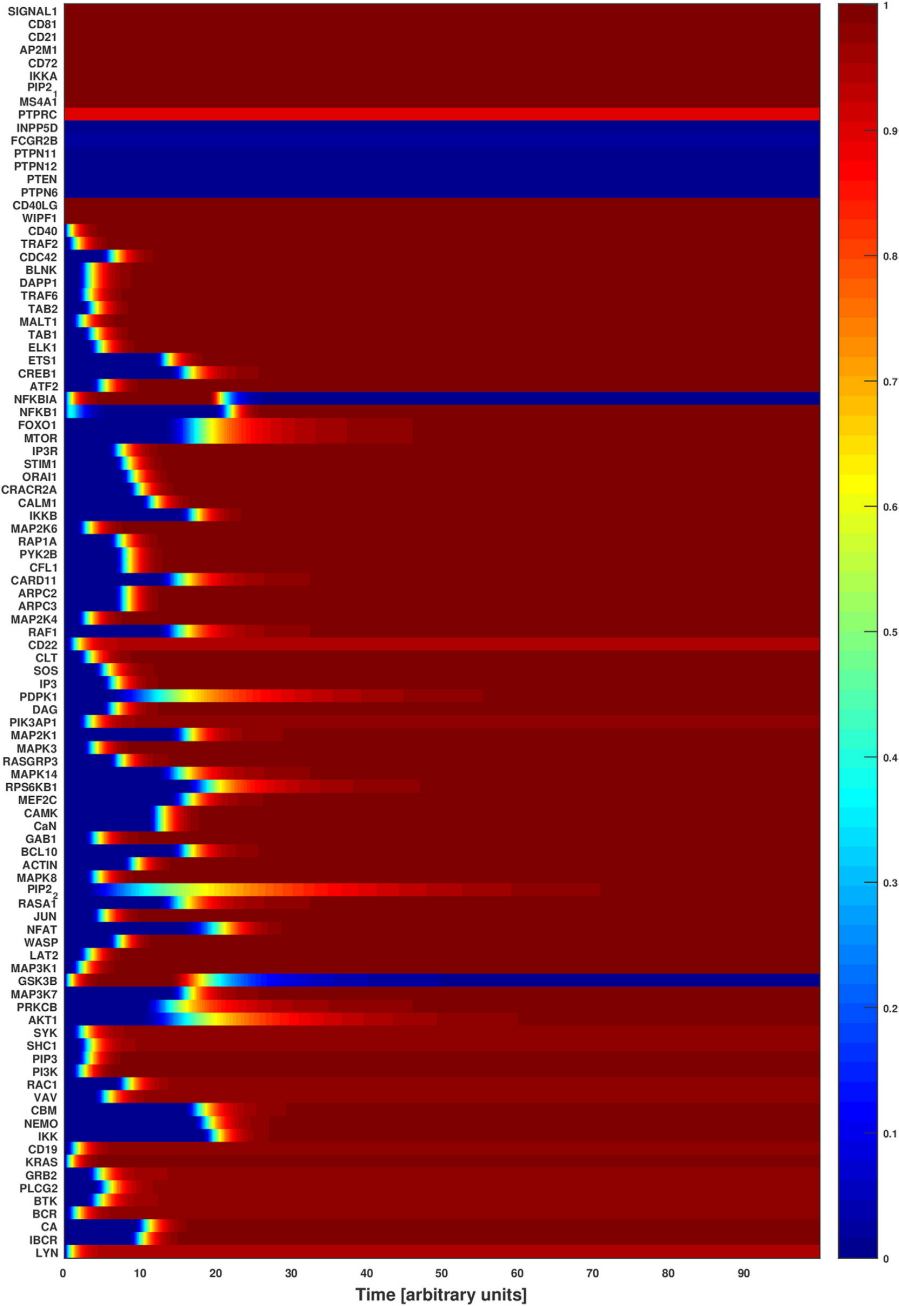
Attractor basin of the B cell network model’s normalized HillCube simulation. The basin of attractors of the B cell network model was simulated using the normalized HillCube algorithm. The horizontal axis denotes updates in time steps in arbitrary units. The simulation was run until the network reached a point attractor (the activating attractor) after about 80 time steps. At time points 110–210 the modulators of the B cell receptor signaling events (INPP5D, FCGR2B, PTPN6, PTPN11, PTPN12, PTEN, and HDAC4) were transiently activated. The network reached another attractor (the modulating attractor) at around time point 230. The simulations then continued until the activating attractor was reached again around time point 290.

We ran the normalized HillCube simulations until the networks reached their attractor states. The signaling events triggered by BCR activation were run until the network settled in an attractor after about 80 updates. The resulting attractors were in accordance with published experimental results (3, 41). For example, upon BCR stimulation, a cascade of phosphorylation leads to the activation of Src kinases (LYN, BLK, or FYN), SYK, BLINK BTK, PLCG2, and other proteins that form the early activation complex, which in turn leads to the calcium signaling (42). The activation of these proteins is captured in the basin of attraction of Figure 3. The simulations indicate also the activation and regulation of the major downstream TFs (ATF2, CREB1, ELK1, ETS1, FOXO1, JUN, MEF2C, NFKB1, and NFAT) when the signals 1 and 2 are turned on. In conclusion, the simulated network transmits signals to the TFs when both signals 1 and 2 are on, just as expected. Perturbations of key factors impair this flow of information and indicate disease-related processes due to defective signaling.

### PID Failure Analysis

We studied the effects of disease-causing variations on the long-term dynamics of B cells by perturbing PID proteins in the network model. Twenty-three PIDs are known to affect the proteins in the network including BCL10, BLNK, BTK, gain-of-function (GOF), and loss-of-function CARD11, CD19, CD21, CD40, CD81, IKKB, KRAS, LYN, MALT1, MS4A1, NEMO, NFKB1, NFKBIA, ORAI1, PI3K, PLCG2, PTPRC, STIM1, and WIPF1 deficiencies. Although no case of LYN deficiency has been reported, LYN was studied to represent PIDs connected to the Src-family kinases (SFKs). Thus, BLK was represented by LYN in the network model. To the best of our knowledge, BLK is the only SFK involved in B cell PIDs (43). The known PID proteins were identified from the ImmunoDeficiency Resource (44), IDbases (8), the classification by the IUIS expert committee for PIDs (9), and a recent review (45). These proteins are expressed at different stages during the B cell development. Here, we focused on PIDs that occur during pre- and mature B cell developmental stages. The effects of complete knockouts or knockins of these proteins were investigated by turning them off or on during simulations, respectively. The resulting perturbed attractors were compared with that of the wild type.

**Figure 4:**
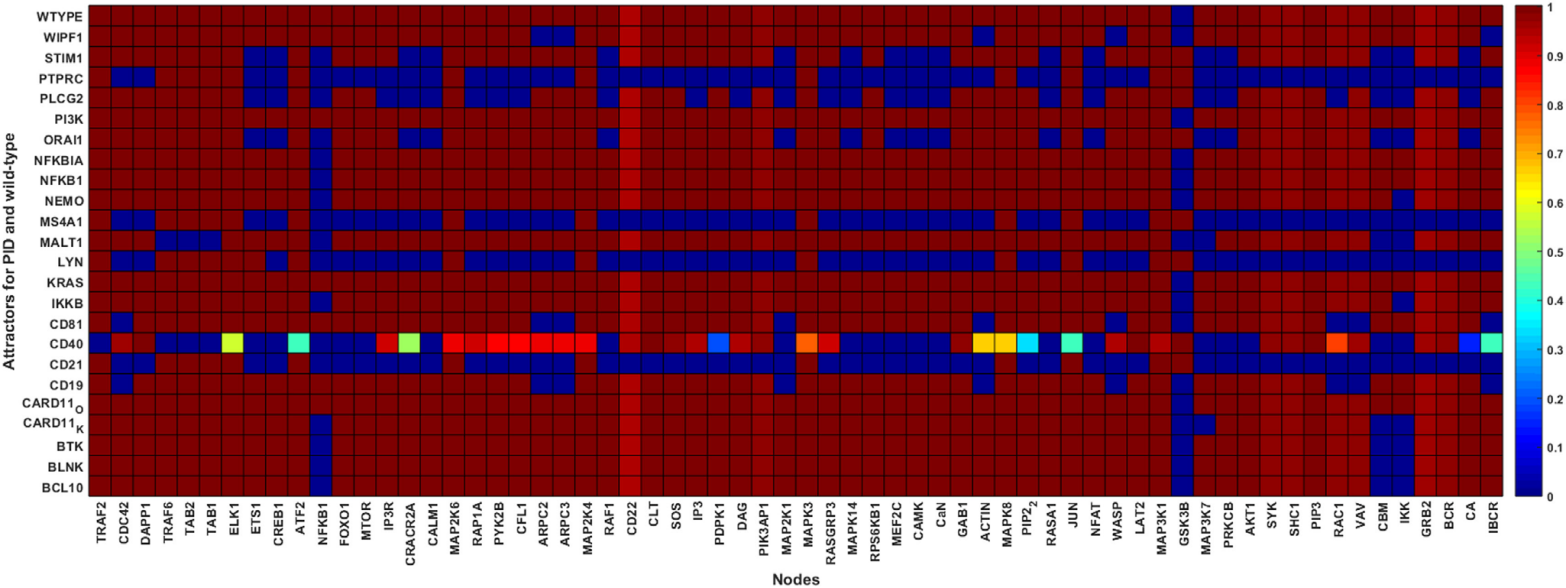
Wild-type and primary immunodeficiency (PID) attractors of the B cell network simulation. The node states for the wild-type and the PID-perturbed attractors (knockout perturbation of BCL10, CARD11 loss-of-function, CD19, CD21, CD40, CD81, IKKB, LYN, MALT1, MS4A1, NEMO, NFKB1, NFKBIA, ORAI1, PI3K, PLCG2, STIM1, and WIPF1 and knockin perturbation of CARD11 gain-of-function, KRAS, NFKBIA, and PI3K) attractors. The attractors are represented by the rows while the states of the nodes in the attractors are represented on the columns. The state of a node for an attractor is represented by the color of the cell on the row of the attractor; black means inactive whereas white means active. The lighter the color of the cell, the more active the protein.

None of the major TF signaling pathways were disrupted in the CARD11, KRAS, and PI3K overexpression PID attractors, and CD19, CD81, and WIPF1 knockout attractors (Figure 4). Several TF pathways were dysregulated in the attractors of PIDs involved in the early events of the BCR-dependent B cell activation including BLNK, BTK, CD21, CD40, LYN, MS4A1, and PTPRC (Figure 4). The perturbations of most of the PIDs indicate profound effects in the pathways essential for B cell survival and response. In the wild-type attractor, the ETS1 TF is turned on during the simulation. This is in accordance with its inhibitory role in BCR response (46). Furthermore, ETS1-deficient mice show enhanced expression of activating markers and increased secretion of autoantibodies (47). However, ETS1 is turned off in the CD21, CD40, MS4A1, ORAI1, PLCG2, PTPRC, and STIM1 PID attractors. CREB1 is associated with antigen-BCR-dependent survival signals. The activating TFs CREB1, MEF2C, and NFAT pathways were dysregulated in the CD21, CD40, LYN, MS4A1, ORAI1, PLCG2, PTPRC, and STIM1 deficiency attractors. Except for CD19, CD18, and WIPF1 PID attractors, NF-κB signaling pathway was disrupted in all PID attractors. FOXO1, a critical downstream effector of the PI3K/AKT signal transduction axis involved in controlling growth arrest and apoptosis, was turned on in the wild-type simulation, as verified by experiments (48). In the PID simulations, the characteristics of FOXO1 are similar to that of the wild type except for CD21, CD40, LYN, MS4A1, and PTPRC PID attractors. ELK1, ATF2, and JUN pathways are partially dysregulated in the CD40 deficiency attractors, and normal in all other PID attractors. These TFs are phosphorylated and activated by the MAPK signaling relay and are associated with BCR-dependent survival signals (3).

The simulation results agree with experimental evidence. We have chosen examples from the different parts of the network. CD40, variants which can lead to hyper-IgM syndrome (HIGM3) (49, 50) due to impaired somatic hypermutation generating antibody variation, provides one of the central inputs for B cell activation. In our simulations, CD40 defect has a profound effect on the entire network in line with its central role for BCR signaling. BTK is in the middle of the network. Variants in this protein lead to X-linked agammaglobulinemia due to defective BTK tyrosine kinase activity. Btk is critical for B-cell development, differentiation, and signaling. Harmful BTK variants block B-cell differentiation to pro- and pre-B-cell stages. BTK is involved in calcium mobilization and fluxes, cytoskeletal rearrangements, and transcriptional regulation involving NF-κB (51, 52). The simulation results indicate effects on NF-κB and IKK similar to experimental evidence.

NEMO is an inhibitor of κB kinase gamma and regulates NF-κB by phosphorylating IκB leading to degradation of it and subsequent activation of NF-κB (53). Thus, the results indicating effects on NFKB1 and IKK agree with experimental studies. Defective NEMO signaling causes X-linked hypohidrotic ectodermal dysplasia with immunodeficiency and other diseases including osteopetrosis and lymphedema.

### Correlation With PID Severity

Primary immunodeficiencies differ greatly in severity from very mild to life-threatening conditions. Severe combined immunodeficiency (SCID) is associated with high vulnerability to infectious diseases and can be lethal (54). According to the IUIS classification (9), some of the PIDs in this study are associated with SCIDs with reduced numbers or absent T and B cells subtypes or isotypes. These include BCL10, CARD11, CD40, IKKB, MALT1, and PTPRC deficiencies. The attractors of these PIDs show less dysregulation except for CD40 (Figure 4). However, most of the PIDs disrupt the NF-κB pathways.

Combined immunodeficiencies (CIDs) are less severe compared with SCIDs and have variable clinical phenotypes. NEMO, GOF NFKBIA, ORAI1, STIM1, and WIPF1 constitute CIDs. Most of these proteins are components of calcium signaling, and impair, as expected, the NF-κB and NFAT pathways, both of which are activated by calcium signaling.

Gain-of-function variants in the *PIK3CB* gene that codes for the catalytic subunit of the PI3K heterodimeric complex are associated with a mild PID (55–57). In addition, variants in the gene that code for NFKBIA are associated with various forms of ectodermal dysplasia with immunodeficiency (58–61). The attractors for both PI3K and NFKBIA are similar to the wild-type indicative of a mild effect.

Many B cell PIDs are antibody deficiencies and have less severe but recurrent infections. These include BTK, BLNK, CARD11, CD19, CD21, CD81, PI3K, and STIM1 deficiencies. These PIDs show minor alterations to the investigated network, except for CD21 and STIM1. Nonetheless, the NF-κB pathway is disrupted in most of these PID simulations, as is the case with the experimental studies. Altogether, the PID severity analysis verifies that proteins involved in severe diseases are central for the network.

### Novel PID Candidate Proteins

New PIDs are still being discovered, but due to their large number, rarity and overlapping symptoms, their diagnosis may be late, challenging and costly. Several classifications of PIDs have been introduced (9, 10), and candidate genes and proteins have been suggested (22, 62–65). Proteins that affect several pathways and are captured by the signaling loops are likely involved in PIDs. Proteins that appear in at least 10% of the loops include the majority of the investigated PIDs (15 of 22) and several proteins essential for B cell activation and functions. Interestingly, some of these proteins also indicate abrogated signaling in the attractors for most of the PIDs. To evaluate *in silico* the effects of perturbing proteins that have not, thus far, been experimentally connected to PIDs in Table 2, we performed knockout simulations for each of them. All the perturbed nodes impaired BCR-dependent survival and response signaling. Moreover, we analyzed from the Human Gene Connectome (HGC) (66) web server biologically and functionally close genes for known PIDs and found connections between proteins present in numerous loops and significant associations to known PID proteins (Table 2). Among these are SYK, PRKCB, IP3R, and GAB1 that are important for BCR activation and survival response. In addition, many of our candidate genes have been suggested in other studies (62, 63).

**Table 2:** Novel primary immunodeficiency (PID) candidates.

Protein	% FFLs^a^	Human Gene Connectome BRP	Itan and Casanova (*p*-value)	Keerthikumar et al. (*p*-value)
SYK	22.63	0.001310	0.00007	4.24
PRKCB	14.06	0.000780	0.00042	3.70
IP3R	41.25	0.000006	0.00113	NA
GAB1	21.11	0.001140	0.00149	3.86
LAT2	56.25	0.015470	0.00205	0.88
GRB2	31.84	0.002210	0.00014	2.13
SHC1	36.38	0.002210	0.00007	NA
MAP3K1	18.85	0.000720	0.00057	NA
MAPK14	10.79	0.001430	0.00064	0.94
MAPK3	36.38	0.002330	0.00057	4.5
PIK3AP1	10.79	0.000180	NA	3.1
MAP2K1	15.00	0.000720	0.00354	3.34
RAF1	75.42	0.002030	0.00007	4.45
MAP3K7	36.25	0.002810	0.00021	2.52
RAC1	11.34	0.010990	0.00007	4.30
CRACR2A	16.42	0.002750	NA	NA
MAP2K4	71.00	0.004060	0.00318	1.34

*Columns 4 and 5 consist of *p*-values of novel candidate PIDs scores for the proteins from Itan and Casanova (62) and Keerthikumar et al. (63), respectively*.

*^a^The fraction of FFLs that the protein is part of. There were 6,542 such loops*.

## Discussion

In this study, we reconstructed the B cell network model based on the literature, refined and *in silico* validated it, and then used it to study the semi-quantitative dynamic effects of PID knockouts and knockins. The model captures the main BCR activation signaling components in B cells. The normalized HillCube approach in the Odefy toolbox was used to simulate both the normal and the PID perturbations in the model for the B cell network. The perturbations qualitatively replicated experimental data for several PIDs at the pre- and mature B cell developmental stages.

The validity of the network and our approach were tested at several levels. First, the signal transduction from the BCR to TFs was shown to be intact and affected by perturbations of the proteins. Second, in many PIDs proteins were shown to be highly connected and involved in numerous signaling loops. Third, the resulting attractors from PID simulations were in line with experimental results for diseases in these proteins. Fourth, severity of several PIDs correlates with effects to information flow in the network. In summary, the network and simulations capture important characteristics of PIDs and can thus be used to extrapolate to other proteins in the network.

We compared the wild-type to the PID attractors and found severe signal transduction defects when CD21, CD40, LYN, MS4A1, ORAI1, PLCG2, PTPRC, and STIM1 were perturbed. The trends that indicate major changes in signaling patterns were captured in the knockout simulations. Minor differences were observed between the wild-type and CARD11, KRAS, and PI3K overexpression attractors, and CD19, CD81, and WIPF1 knockout attractors. The pathway for NF-κB was disrupted in all the PID knockouts except in the CD19, CD81, and WIPF1. These proteins connect receptor-dependent signals to the distal NF-κB pathway (67). knockout of any of these proteins may impair the IKK complex, the major NF-κB regulator, by leaving NFKBIA bound to NFKB1, thereby preventing its nuclear transportation and function as a transactivator (67).

After BCR-antigen activation, B cells undergo somatic hypermutation and antibody class switching. ELK1, ATF2, and JUN are effectors downstream to the RAS/MAPK pathways in the BCR signaling network. They control survival, differentiation and proliferation responses in B cells after activation (31). These TFs were not affected except (slightly) in the CD40 PID attractor. The ETS1 TF controls survival and differentiation, and thus its activity is increased through calcium ion signaling during BCR signaling (32, 46, 47, 68). In our simulations, the PID attractors for proteins involved in calcium signaling disrupted the ETS1 pathway. All other TFs that regulate survival and proliferation signals through the BCR signaling were abrogated in at least five PID attractors (Figure 4). These results verify that our simulation approach is effective when the affected proteins are at the core of the interconnected network or along non-redundant paths. No major changes were revealed in overexpression perturbations, as in PI3K, GOF CARD11, KRAS, and NFKBIA disorders. All the PID proteins, excluding those at input nodes, emerge in the signaling loops. LYN, STIM1, ORAI1, and PLCG2 perturbations, which cause major effects, are present in over two-thirds of the loops.

Our results show PID trends in the cellular dynamics of the B cells when the affected proteins are involved in non-redundant paths along the major TF signaling pathways. Perturbations of early events show more profound network disruption than those affecting late events. This is demonstrated in the profound outcomes when perturbing PID proteins taking part in the early events (CD21, CD40, CD81, LYN, MS4A1, and PTPRC), and less profound, but noticeable effects, in the intermediate and late events (IKKB, ORAI1, PLCG2, and STIM1).

The proteins in Table 2 that are not linked to known PIDs are essential for B cell activation and function and are affected in several B cell simulated attractors. Many of them have been previously identified as PID candidates. *GAB1, GRB2, LAT2, MAP2K1, MAP3K7, MAPK14, MAPK3, MAPK8, PIK3AP1, PRKCB, RAF1*, and *SYK* have been designated as candidate PID genes (63). Fourteen out of these 17 proteins were predicted as PID candidates in another study (62) including IKKA, GAB1, GRB2, LAT2, MAP2K1, MAP3K7, MAPK14, MAPK3, MAPK8, PIK3AP1, PRKCB, RAF1, and SYK. Several of the candidate genes are connected to PID proteins in the HGC (Table 2), providing independent proof for their significance.

According to the Panther databases (69), eight of the proteins in Table 2 are non-receptor protein kinases (GRB2, IKKA, MAP3K7, MAPK3, MAPK8, MAPK14, RAF1, and SYK), three are mitogen-activated and phosphotyrosine binding kinases (MAPK3, MAPK14, and RAF1), four have calcium ion binding activity (ITPR1, LAT2, MAP3K7, and PRKCB), and one has SH3/SH2 adaptor activity (GAB1). According to the Genecards and Malacards suites (70, 71), eight of the proteins are associated with various cancer types (GRB2, MAPK3, MAPK8, MAPK14, PRKCB, RASA1, SHC1, and SYK), four to different forms of cardiovascular diseases (MAP2K1, RAF1, RASA1, and SHC1), three to various Noonan syndromes (GAB1, MAP2K1, and RAF1), two to liver diseases (MAPK8 and SHC1), two to neuroendocrine neoplasm (MAPK3 and MAPK8), and one each to inflammatory bowel disease (SHC1), ectodermal dysplasia (IKKA), and Wiskott-Aldrich syndrome (GRB2). These proteins or their encoding genes could also have effects on PIDs. The listed proteins are strong PID candidates; however, their involvement in PIDs is yet to be experimentally verified.

Primary immunodeficiency candidate genes have been proposed in several studies (62–64). With reconstructed PPI network of immune system-specific proteins, proteins with high network statistics and PID-associated Gene Ontology term enrichment scores were considered as candidates (64). Itan and Casanova identified the top 1% of genes biologically close to known PIDs and selected those with similar Gene Ontology terms as the known PIDs (62). Support vector machine, a supervised machine learning technique, has been used to identify candidate PIDs (63). Several of the detected candidate genes above have been later confirmed to be PID-associated. Our approach focuses on B cell-specific network model, B cell intrinsic PIDs, the proteins that are connected to the PIDs and their effects on the BCR signaling *in silico*.

Differential diagnosis and treatment of PIDs is still challenging. Our approach provides novel insight to the mechanisms of PIDs in immune response signaling and presents new candidates for therapy and diagnosis. Experiments to validate the proposed PID candidates are still to be conducted. Furthermore, detailed quantitative models of the knockin perturbations would shed more light on their effects and require more detailed experimental data.

## Materials and Methods

### Network Reconstruction and Analysis

The B cell network model was reconstructed through extensive literature mining. We focused on the major components required for BCR/CD40-dependent B cell activation signaling events. Furthermore, we included only signaling events for undifferentiated B cells. Boolean equations were constructed based on literature evidence. The network is available in SBML format (Table S3 in Supplementary Material) and at the NDEx network provenance repository (72) with the UUID: *2554db2d-7533-11e8-a4bf-0ac135e8bacf*. The reconstructed Boolean network model was used for wild-type and PID-perturbed simulations using Odefy, a Matlab toolbox (19), with the normalized HillCube method (73). NetDS, a Cytoscape plugin (74), was used for identifying signaling loops in the underlying interaction graph of the model. Data analysis was done with the R software, version 3.2.3 (75), and *igraph*, a library for network and graph analyses in R (76). Cytoscape, version 3.5.1 (77) was used for network visualization.

The analyses and simulation protocol were essentially similar to what we used to investigate T-cell PIDs (22). Briefly, in the Boolean model the nodes represent *N* signaling molecule or protein variables, *X*_1_, *X*_1_, …, *X_N_*. The value of a variable is either 0 or 1 (78). Proteins, *x*_i_, are influenced by their neighbors, *R_i_* = {*X*_1_, *X*_1_, …, *X_N_*}. The value of a protein at time *t* is updated at time *t* + 1 from the values of its neighbors, *R_i_*, with update function *B*: {0, 1*^N^*}. The discrete and synchronous update (78, 79) was performed according to the following equation:

$$x_{i}\left(t+1\right)=B_{i}\left(x_{i1}\left(t\right),x_{i2}\left(t\right),\ldots ,x_{iN_{i}}\left(t\right)\right)\text{ ε }\{0,1\},\;i=1,2,\ldots ,N.$$

The ordinary differential equation (ODE) equivalent of the Boolean update functions, where *x_i_* takes values [0, 1] was computed using

$${\dot{x}}_{i}=\frac{1}{{\text{τ}}_{i}}\left({\bar{B}}_{i}({\bar{x}}_{i1},{\bar{x}}_{i2},\ldots ,{\bar{x}}_{iN_{i}}\right)-{\bar{x}}_{i}),$$

where ${\bar{B}}_{i}$ is a continuous homolog of the discrete function *B_i_*, parameter τ*_i_* is the life-time of the protein, and ${\bar{x}}_{i}$ describes its decay.

Odefy (19) transforms the discrete Boolean to the continuous system of ODEs and computes the solution of the system using the BooleCubes (73) as follows:

$${\bar{B}}^{I}\left({\bar{x}}_{1},{\bar{x}}_{2},\ldots ,{\bar{x}}_{N}\right)=\sum_{x_{1}=0}^{1}\sum_{x_{1}=0}^{1}\ldots \sum_{x_{N}=0}^{1}\;[B\left(x_{1},x_{2},\ldots ,x_{N}\right).\prod_{i=1}^{N}\left(x_{i}{\bar{x}}_{i}+\left(1-x_{i}\right)\left(1-{\bar{x}}_{i}\right)\right)].$$

${\bar{B}}^{I}$, the BooleCube, is obtained from interpolating *B_i_*, the Boolean discrete update function. Since molecular interactions are not switch-like in behavior, the Hill function,

f(x¯)−x¯n(x¯n−k¯n),

was applied to the BooleCube to obtain a sinusoidal function, the HillCube (73) as follows, ${\bar{B}}^{H}({\bar{x}}_{1},\ldots ,{\bar{x}}_{N})={\bar{B}}^{I}(f_{1}({\bar{x}}_{1}),\ldots ,f_{N}({\bar{x}}_{N}))$.

The parameter *n* (the Hill coefficient) represents the cooperativity between the protein interactions, while parameter *k* represents the value at which the activation is half-maximal.

To obtain perfect homologs of the Boolean update functions *B_i_*, the HillCube functions were normalized to the unit interval to give the normalized HillCube (73) as follows, ${\bar{B}}^{Hn}({\bar{x}}_{1},\ldots ,{\bar{x}}_{N})={\bar{B}}^{I}\left(\frac{f_{1}({\bar{x}}_{1})}{f_{1}(1)},\ldots ,\frac{f_{N}({\bar{x}}_{N})}{f_{N}(1)}\right)$.

### Basin of Attraction and Attractor Identification

The Odefy toolbox was used to simulate the qualitative dynamics of the network model (19). We used the normalized HillCube functions with synchronous updates. The default parameters for the normalized HillCube were *n* = 3, *k* = 0.5, and τ = 1. Except for the nodes in Table 1, the default parameter values were used. The variable *n* represents the Hill exponent of the Hill function and is used for converting the discrete Boolean update functions that take value 0 or 1 into their continuous BooleCube equivalents that have values [0, 1]. It captures the influence that the nodes of the same Boolean equation have on each other. *k* is a variable to control the continuous relaxation of the Boolean step function. It represents the value at half-maximal activation of a protein. τ is a decay parameter; for each signaling molecule, the higher its value, the slower the decay of the molecule.

The parameters in Table 1 were adjusted so that the wild-type attractor represents experimental data from the literature. The simulations were run until the network dynamics settled in the attractor that captures BCR activation.

### Perturbation

Analysis of PID effects was performed for each protein encoded by a PID gene using the normalized HillCube simulations. For each perturbation, the node was converted to an input before assigning a state, either off or on, depending on the PID. This state was maintained until the simulation transitioned into the attractor. The parameter values used in the wild-type simulations were maintained for all the PID-perturbed simulations. The result of the simulation is the perturbed PID attractor.

### Primary Immunodeficiencies

Primary immunodeficiency proteins expressed in B cells were retrieved from the IDbases (8, 44), the updated IUIS expert committee classification of PIDs (9), and a recent survey (45), and used for the PID failure mode simulations. The studied PIDs included BCL10, BLNK, BTK, CARD11, CD19, CD21, CD40, CD81, IKKB, KRAS, LYN, MALT1, MS4A1, NEMO, NFKB1, NFKBIA, ORAI1, PI3K, PLCG2, STIM1, and WIPF1 deficiencies.

## Author Contributions

GT designed and developed the systems biology approach, interpreted data, performed data processing, interpreted the results, and wrote the manuscript. MV designed the research question, interpreted data, corrected the manuscript, and supervised the work. GT and MV have read and approved the final version of the manuscript.

## Conflict of Interest Statement

The authors declare that the research was conducted in the absence of any commercial or financial relationships that could be construed as a potential conflict of interest.

## Supplementary Material

Table S1 B cell activation Boolean network model update equations. The table lists Boolean equations of protein activation used in the network model and simulations.

Table S2 Signaling loops from the B cell interaction network of the Boolean network. The table consists of the number signaling loops that each node was a part of. All the loops included primary immunodeficiency proteins. All input nodes were excluded from the table. Signal1 (antigen) and CD40LG (Signal 2) were the source of all loops, and the rest of the input signals did not occur in any loop.

Table S3 SBML qual. The B cell network model in SBML qual format.
