# Supplementary material for: Simulation of the Dynamics of Primary Immunodeficiencies in B Cells
Source: Front Immunol. 2018 Aug 2;9:1785. doi: 10.3389/fimmu.2018.01785 (PMC6082931; doi:10.3389/fimmu.2018.01785)
Supplement: Table S1 — B cell activation Boolean network model update equations. The table lists Boolean equations of protein activation used in the network model and simulations. [file Table_1.docx]

# Table of activated targets by interacting effectors

| **Targets** | **Factors** | **References** |
| --- | --- | --- |
| SIGNAL1 | INPUT SIGNAL | (Dal Porto et al., 2004) |
| DOK3 | INPUT SIGNAL | (Manno et al., 2016) |
| AP2M1 | INPUT SIGNAL | (Lu et al., 2002) |
| EZR | INPUT SIGNAL | (Pore & Gupta, 2015) |
| CD72 | INPUT SIGNAL | (H. J. Wu & Bondada, 2009) |
| CD40 | INPUT SIGNAL | (Francis, Karras, Ke, Sen, & Rothstein, 1995) |
| ACTIN | INPUT SIGNAL | (J. C. Wang et al., 2017) |
| TRAF2 | INPUT SIGNAL | (Hacker & Karin, 2006; Meininger & Krappmann, 2016; Mitchell, Vargas, & Hoffmann, 2016) |
| IKKA | INPUT SIGNAL | (Hacker & Karin, 2006) |
| SIGNAL2 | INPUT SIGNAL | (Brunswick, June, & Mond, 1994) |
| PIP2_1 | INPUT SIGNAL | (Saito et al., 2003) |
| MS4A1 | INPUT SIGNAL | (Leandro, 2013) |
| PTPRC | INPUT SIGNAL | (Pao, Bedzyk, Persin, & Cambier, 1997a) |
| INPP5D | INPUT SIGNAL | (Aman, Lamkin, Okada, Kurosaki, & Ravichandran, 1998) |
| FCGR2B | INPUT SIGNAL | (Dal Porto et al., 2004) |
| PTPN11 | INPUT SIGNAL | (Ingham et al., 2001; Maeda, Kurosaki, Ono, Takai, & Kurosaki, 1998) |
| PTPN12 | INPUT SIGNAL | (Ingham et al., 2001; Maeda et al., 1998) |
| HDAC4 | INPUT SIGNAL | (Weiss et al., 2003) |
| PTEN | INPUT SIGNAL | (Maehama & Dixon, 1998) |
| PTPN6 | INPUT SIGNAL | (Ingham et al., 2001; Maeda et al., 1998) |
| PAG1 | LYN | (Ingley et al., 2006) |
| CSK | PAG1 & DOK3 | (Brdicka et al., 2000; Kawabuchi et al., 2000; Lemay, Davidson, Latour, & Veillette, 2000) |
| CDC42 | VAV | (O'Rourke et al., 1998; Turner & Billadeau, 2002) |
| BLNK | SYK & ! PTPN6 | (Fu, Turck, Kurosaki, & Chan, 1998; Ishiai et al., 1999; Mizuno et al., 2000) |
| DAPP1 | PIP3 & SYK | (Anderson et al., 2000; Stephens, Anderson, & Hawkins, 2001) |
| RHOA | NCK & PIP3 | (Homma & Emori, 1995) |
| BAD | ! AKT1 | {Thompson, 2004 #524} |
| TRAF6 | MALT1 | (Hacker & Karin, 2006; Meininger & Krappmann, 2016) |
| TAB2 | TRAF6 | (Hacker & Karin, 2006; Meininger & Krappmann, 2016) |
| MALT1 | TRAF2 & TRAF6 | (Z. J. Chen, 2012; King et al., 2006; Paul & Schaefer, 2013; Thome, 2004) |
| TAB1 | TRAF6 | (Hacker & Karin, 2006; Meininger & Krappmann, 2016) |
| BCL6 | MAPK3 | (Hatzi & Melnick, 2014) |
| EGR1 | MAPK3 | (Richards, Dave, Chou, Mamchak, & DeFranco, 2001) |
| ELK1 | MAPK3 | (Koncz, Bodor, Kovesdi, Gati, & Sarmay, 2002) |
| BCLXL | ! BAD | (Niiro et al., 2012) |
| ETS1 | ! CAMK | (Valentine et al., 1995) |
| CREB | MAPK14 | (Swart, Bergeron, & Chiles, 2000) |
| ATF2 | MAPK8 | (S. Gupta, Campbell, Derijard, & Davis, 1995) |
| NFKBIA | ! IKK | (Hayden & Ghosh, 2004) |
| NFKB1 | ! NFKBIA | (Hayden & Ghosh, 2004) |
| FOXO1 | ! AKT1 | (Yusuf, Zhu, Kharas, Chen, & Fruman, 2004) |
| MTOR | AKT1 | (Majchrzak, Witkowska, & Smolewski, 2014) |
| CD81 | CD19 | {Sato, 1997 #342} |
| CD21 | CD19 | (Sato, Miller, Howard, & Tedder, 1997; Tedder, Inaoki, & Sato, 1997) |
| IP3 | PLCG2 & PIP2 & X | (Justement, Campbell, Chien, & Cambier, 1991; Justement, Wienands, Hombach, Reth, & Cambier, 1990; Saunders & Johnson, 2010) |
| IP3R | IP3 | (Lyubchenko, 2010) |
| STIM1 | IP3R | (Lyubchenko, 2010) |
| ORAI1 | STIM1 | (Lyubchenko, 2010) |
| CRACR2A | ORAI1 | (Lyubchenko, 2010) |
| CALM1 | CA | (Gallo, Cante-Barrett, & Crabtree, 2006) |
| IKKB | MAP3K7 & TAB2 & TAB3 | (Meininger & Krappmann, 2016; C. Wang et al., 2001) |
| MAP2K6 | MAP3K1 | (Yasuda, 2016) |
| RAP1A | DAG | (McLeod & Gold, 2001) |
| PYK2B | RAP1A & CSR | (Tse et al., 2009) |
| CFL1 | RAP1A | (J. C. Wang et al., 2017) |
| RKS6KB1 | PDPK1 | (Vega et al., 2006) |
| CARD11 | PRKCB & MAP3K7 | (Shinohara et al., 2005) |
| ARPC2 | WASP | (Song et al., 2013) |
| ARPC3 | WASP | (Song et al., 2013) |
| RAF1 | KRAS | (Kawauchi et al., 1996) |
| CD22 | LYN | (Cornall et al., 1998; Smith, Tarlinton, Doody, Hibbs, & Fearon, 1998; Tedder, Poe, & Haas, 2005) |
| CLT | SYK && AP2M1 | (Stoddart et al., 2002; Traub, 2003) |
| SOS | GRB2 & LAT2 | (Brdicka et al., 2002; Chardin et al., 1993; Ravichandran, Lorenz, Shoelson, & Burakoff, 1995) |
| NCK | BLNK & SOS | (Fu et al., 1998) |
| PDPK1 | PIP2_2 && PIP3 | (Gratacap et al., 1998; Saito, Scharenberg, & Kinet, 2001) |
| DAG | PLCG2 & PIP2 & RASGRP3 | (Zheng et al., 2005) |
| PIK3AP1 | LYN & SYK | (Okada, Maeda, Iwamatsu, Gotoh, & Kurosaki, 2000) |
| MAP2K1 | RAF1 & CD19 | (Kawauchi et al., 1996; Kyriakis et al., 1992) |
| MAPK3 | MAP2K1 & CD40 | (Sutherland, Heath, Pelech, Young, & Gold, 1996) |
| RASGRP3 | DAG | (Ehrhardt, David, Ehrhardt, & Schrader, 2004; Lorenzo et al., 2001; Zheng et al., 2005) |
| WIPF1 | WASP && ACTIN | (Arana et al., 2008) |
| MAPK14 | MAP2K6 & PRKCB | (Cuenda, Cohen, BueeScherrer, & Goedert, 1997; Doza, Cuenda, Thomas, Cohen, & Nebreda, 1995; Enslen, Raingeaud, & Davis, 1998; Goedert, Cuenda, Craxton, Jakes, & Cohen, 1997; A. Lin et al., 1995) |
| - RPS6KB1 | MTOR | (Vega et al., 2006) |
| MEF2C | MAPK14 & CA | (Blaeser, Ho, Prywes, & Chatila, 2000; Zhao et al., 1999) |
| CAMK | CALM1 & CA | (Gallo et al., 2006) |
| CaN | CALM1 & CA | (Gallo et al., 2006) |
| GAB1 | BCR & PTPN11 & LAT2 | (Brdicka et al., 2002; Ingham, Holgado-Madruga, Siu, Wong, & Gold, 1998; Ingham et al., 2001) |
| BCL10 | TRAF6 & LUBAC & CARD11 | (King et al., 2006; Paul & Schaefer, 2013; Thome, 2004) |
| MAPK8 | (MAP2K4 \| MAP2K7) & CA & PRKCB | (Yasuda, 2016) |
| PIP2 | PIP5K & RHOA | (Homma & Emori, 1995; Saito et al., 2003) |
| RASA1 | PRKCB & DOK1 & FCG2RB | (Lorenzo et al., 2001; Tamir et al., 2000) |
| JUN | MAPK8 & ! HDAC4 | (Fuchs, Dolan, Davis, & Ronai, 1996; Sabapathy et al., 2004; Weiss et al., 2003) |
| NFAT | CaN & ! GSK3B | (Beurel, Michalek, & Jope, 2010; Gallo et al., 2006) |
| WASP | CDC42 \|\| RAC1 | (Arana et al., 2008) |
| LAT2 | GRB2 & SYK | (Arana et al., 2008; Brdicka et al., 2002; Iwaki, Jensen, & Gilfillan, 2007; Koonpaew, Janssen, Zhu, & Zhang, 2004) |
| MAP3K1 | RAC1 \| CDC42 | (Han et al., 1998; Yasuda, 2016) |
| GSK3B | ! AKT1 \| ! PRKCB | (Astoul, Watton, & Cantrell, 1999; Christian, Sims, & Gold, 2002; Gold et al., 2000; Gold et al., 1999) |
| MAP3K7 | TAB1 && TAB2 && CARD11 | (Shinohara et al., 2005; C. Wang et al., 2001) |
| PRKCB | DAG & CA | (Kawauchi et al., 1996; Lorenzo et al., 2001; Stone, 2011) |
| DOK1 | FCGR2B && INPP5D && LYN | {Mashima, 2009 #525} |
| AKT1 | PIP3 & ! INPP5D | (Aman et al., 1998; Datta et al., 1995) |
| SYK | BCR & ! PTPN6 & PTPRC & ! CSK & ! (CBLB \| CBL) | (Huang & Gu, 2008; Katkere, Rosa, & Drake, 2012; Niiro et al., 2012; Pao, Bedzyk, Persin, & Cambier, 1997b; Pao & Cambier, 1997; Sohn, Gu, & Pierce, 2003) |
| SHC1 | BCR \| SYK | (Nagai, Takata, Yamamura, & Kurosaki, 1995; Pao, Famiglietti, & Cambier, 1998) |
| PIP3 | PI3K & PIP2 & ! PTEN & ! INPP5D | (Deane & Fruman, 2004; N. Gupta et al., 1999; Leung, Tarasenko, & Bolland, 2009; Maehama & Dixon, 1998) |
| PI3K | PIK3AP1 \| CD19 ) & ! LYN & GAB1 & PIP2) \| (CD40L & CBLB) | (Buhl & Cambier, 1999; Ingham et al., 2001; O'Rourke et al., 1998; Okada et al., 2000) |
| RAC1 | VAV & GRB2 & SOS & (BLNK \| SHC2) & RAP1A | (Arana et al., 2008; D'Ambrosio, Hippen, & Cambier, 1996; Hashimoto et al., 1998; Nagai et al., 1995) |
| VAV | CD19 & ! PTPN6 & GRB2 & PIP3 & LAT2 | (Caloca, Zugaza, Matallanas, Crespo, & Bustelo, 2003; Han et al., 1998; Malhotra, Kovats, Zhang, & Coggeshall, 2009) |
| CBM | CARD11 && MALT1 && BCL10 && MAP3K7 | (Meininger & Krappmann, 2016) |
| NEMO | CBM & BCL10 & MALT1 & SQSTM1 | (Hayden & Ghosh, 2004; Meininger & Krappmann, 2016; C. J. Wu & Ashwell, 2008) |
| IKK | IKKAB & NEMO & MAP3K7 & IKKB | (Hacker & Karin, 2006; X. Lin, Cunningham, Mu, Geleziunas, & Greene, 1999; Meininger & Krappmann, 2016) |
| CD19 | SIGNAL2 & BCR & ! INPP5D & (LYN \| FYN) & CD21 & CD81 | (Chalupny et al., 1995; Cherukuri et al., 2004; Liu et al., 1998; van Noesel, Lankester, van Schijndel, & van Lier, 1993) |
| KRAS | (VAV & GRB2 & SOS & LYN & RHOA) \| RASGRP3 \| RASA1 \| BLNK \| SHC2 | (Caloca et al., 2003; Y. Chen et al., 2016; Chiu, Dalton, Ishiai, Kurosaki, & Chan, 2002; Ehrhardt et al., 2004; Ishiai et al., 1999; Jiang, Craxton, Kurosaki, & Clark, 1998; Oh-hora, Johmura, Hashimoto, Hikida, & Kurosaki, 2003; Tamir et al., 2000) |
| GRB2 | CD22 & CD72 & PIP3 & PTPN11 & DOK3 & SHC1 & LAT2 | (D'Ambrosio et al., 1996; Ingham et al., 2001; Law et al., 1996; Otipoby, Draves, & Clark, 2001; Stork et al., 2007) |
| DARM | WIPF1 && EZR && CFL1 && PYK2B && ACTIN | WIPF1 && EZR && CFL1 && PYK2B && ACTIN |
| PLCG2 | (BLNK \| PIP3 \| PIP2 \| DAPP1) & SYK & BTK & CBLB | (Bae et al., 1998; Chiu et al., 2002; Gratacap et al., 1998; Ishiai et al., 1999; Marshall et al., 2000; Yasuda et al., 2002) |
| BTK | PIP3 & BLNK & SYK & LYN & PRKCB | (Baba et al., 2001; Buhl & Cambier, 1999; Kang et al., 2001; Kurosaki & Kurosaki, 1997; Rawlings et al., 1996; Saito et al., 2001) |
| BCR | SIGNAL1 && LYN && ~PTPN6 && ~PTPN11 && ~FCGR2B | (Daeron, 1997; Flaswinkel & Reth, 1994; Gauld & Cambier, 2004; Liu et al., 1998; Nel, Landreth, Goldschmidtclermont, Tung, & Galbraith, 1984; Pani, Kozlowski, Cambier, Mills, & Siminovitch, 1995; Szydlowski, Jablonska, & Juszczynski, 2014) |
| CA | IP3 & IP3R & STIM1 & ORAI1 & CRACR2A | (Lyubchenko, 2010) |
| IBCR | CLATHRIN & DAPP1 & EZR & RAC1 | (N. Gupta & DeFranco, 2007; Malhotra et al., 2009; Niiro et al., 2004; Stoddart et al., 2002) |
| LYN | BCR & PTPRC & ! CSK & CD19 | (Dal Porto et al., 2004; Ingley et al., 2006; Pao et al., 1997b; Pleiman et al., 1994) |

# References

Aman, M. J., Lamkin, T. D., Okada, H., Kurosaki, T., & Ravichandran, K. S. (1998). The inositol phosphatase SHIP inhibits Akt/PKB activation in B cells. *Journal of Biological Chemistry, 273*(51), 33922-33928.

Anderson, K. E., Lipp, P., Bootman, M., Ridley, S. H., Coadwell, J., Ronnstrand, L., . . . Hawkins, P. T. (2000). DAPP1 undergoes a PI 3-kinase-dependent cycle of plasma-membrane recruitment and endocytosis upon cell stimulation. *Current Biology, 10*(22), 1403-1412.

Arana, E., Vehlow, A., Harwood, N. E., Vigorito, E., Henderson, R., Turner, M., . . . Batista, F. D. (2008). Activation of the small GTPase Rac2 via the B cell receptor regulates B cell adhesion and immunological-synapse formation. *Immunity, 28*(1), 88-99. doi:10.1016/j.immuni.2007.12.003

Astoul, E., Watton, S., & Cantrell, D. (1999). The dynamics of protein kinase B regulation during B cell antigen receptor engagement. *Journal of Cell Biology, 145*(7), 1511-1520.

Baba, Y., Hashimoto, S., Matsushita, M., Watanabe, D., Kishimoto, T., Kurosaki, T., & Tsukada, S. (2001). BLNK mediates Syk-dependent Btk activation. *Proceedings of the National Academy of Sciences of the United States of America, 98*(5), 2582-2586. doi:10.1073/pnas.051626198

Bae, Y. S., Cantley, L. G., Chen, C. S., Kim, S. R., Kwon, K. S., & Rhee, S. G. (1998). Activation of phospholipase C-gamma by phosphatidylinositol 3,4,5-trisphosphate. *Journal of Biological Chemistry, 273*(8), 4465-4469.

Beurel, E., Michalek, S. M., & Jope, R. S. (2010). Innate and adaptive immune responses regulated by glycogen synthase kinase-3 (GSK3). *Trends in Immunology, 31*(1), 24-31. doi:10.1016/j.it.2009.09.007

Blaeser, F., Ho, N., Prywes, R., & Chatila, T. A. (2000). Ca(2+)-dependent gene expression mediated by MEF2 transcription factors. *Journal of Biological Chemistry, 275*(1), 197-209.

Brdicka, T., Imrich, M., Angelisova, P., Brdickova, N., Horvath, O., Spicka, J., . . . Horejsi, V. (2002). Non-T cell activation linker (NTAL): a transmembrane adaptor protein involved in immunoreceptor signaling. *Journal of Experimental Medicine, 196*(12), 1617-1626.

Brdicka, T., Pavilstova, D., Leo, A., Bruyns, E., Korinek, V., Angelisova, P., . . . Schraven, B. (2000). Phosphoprotein associated with glycosphingolipid-enriched microdomains (PAG), a novel ubiquitously expressed transmembrane adaptor protein, binds the protein tyrosine kinase Csk and is involved in regulation of T cell activation. *Journal of Experimental Medicine, 191*(9), 1591-1604. doi:DOI 10.1084/jem.191.9.1591

Brunswick, M., June, C. H., & Mond, J. J. (1994). B-Lymphocyte Immunoglobulin Receptor Desensitization Is Downstream of Tyrosine Kinase Activation. *Cellular Immunology, 156*(1), 240-244. doi:DOI 10.1006/cimm.1994.1168

Buhl, A. M., & Cambier, J. C. (1999). Phosphorylation of CD19 Y484 and Y515, and linked activation of phosphatidylinositol 3-kinase, are required for B cell antigen receptor-mediated activation of Bruton's tyrosine kinase. *Journal of Immunology, 162*(8), 4438-4446.

Caloca, M. J., Zugaza, J. L., Matallanas, D., Crespo, P., & Bustelo, X. R. (2003). Vav mediates Ras stimulation by direct activation of the GDP/GTP exchange factor Ras GRP1. *EMBO Journal, 22*(13), 3326-3336. doi:10.1093/emboj/cdg316

Chalupny, N. J., Aruffo, A., Esselstyn, J. M., Chan, P. Y., Bajorath, J., Blake, J., . . . Tepper, M. A. (1995). Specific Binding of Fyn and Phosphatidylinositol 3-Kinase to the B-Cell Surface Glycoprotein Cd19 through Their Src Homology-2 Domains. *European Journal of Immunology, 25*(10), 2978-2984. doi:DOI 10.1002/eji.1830251040

Chardin, P., Camonis, J. H., Gale, N. W., Vanaelst, L., Schlessinger, J., Wigler, M. H., & Barsagi, D. (1993). Human Sos1 - a Guanine-Nucleotide Exchange Factor for Ras That Binds to Grb2. *Science, 260*(5112), 1338-1343. doi:DOI 10.1126/science.8493579

Chen, Y., Zheng, Y., You, X., Yu, M., Fu, G., Su, X., . . . Wang, D. (2016). Kras Is Critical for B Cell Lymphopoiesis. *Journal of Immunology, 196*(4), 1678-1685. doi:10.4049/jimmunol.1502112

Chen, Z. J. (2012). Ubiquitination in signaling to and activation of IKK. *Immunological Reviews, 246*(1), 95-106. doi:10.1111/j.1600-065X.2012.01108.x

Cherukuri, A., Shoham, T., Sohn, H. W., Levy, S., Brooks, S., Carter, R., & Pierce, S. K. (2004). The tetraspanin CD81 is necessary for partitioning of coligated CD19/CD21-B cell antigen receptor complexes into signaling-active lipid rafts. *Journal of Immunology, 172*(1), 370-380.

Chiu, C. W., Dalton, M., Ishiai, M., Kurosaki, T., & Chan, A. C. (2002). BLNK: molecular scaffolding through 'cis'-mediated organization of signaling proteins. *EMBO Journal, 21*(23), 6461-6472. doi:DOI 10.1093/emboj/cdf658

Christian, S. L., Sims, P. V., & Gold, M. R. (2002). The B cell antigen receptor regulates the transcriptional activator beta-catenin via protein kinase C-mediated inhibition of glycogen synthase kinase-3. *Journal of Immunology, 169*(2), 758-769.

Cornall, R. J., Cyster, J. G., Hibbs, M. L., Dunn, A. R., Otipoby, K. L., Clark, E. A., & Goodnow, C. C. (1998). Polygenic autoimmune traits: Lyn, CD22, and SHP-1 are limiting elements of a biochemical pathway regulating BCR signaling and selection. *Immunity, 8*(4), 497-508.

Cuenda, A., Cohen, P., BueeScherrer, V., & Goedert, M. (1997). Activation of stress-activated protein kinase-3 (SAPK3) by cytokines and cellular stresses is mediated via SAPKK3 (MKK6); Comparison of the specificities of SAPK3 and SAPK2 (RK/p38). *EMBO Journal, 16*(2), 295-305. doi:DOI 10.1093/emboj/16.2.295

D'Ambrosio, D., Hippen, K. L., & Cambier, J. C. (1996). Distinct mechanisms mediate SHC association with the activated and resting B cell antigen receptor. *European Journal of Immunology, 26*(8), 1960-1965. doi:10.1002/eji.1830260842

Daeron, M. (1997). Fc receptor biology. *Annual Review of Immunology, 15*, 203-234. doi:10.1146/annurev.immunol.15.1.203

Dal Porto, J. M., Gauld, S. B., Merrell, K. T., Mills, D., Pugh-Bernard, A. E., & Cambier, J. (2004). B cell antigen receptor signaling 101. *Molecular Immunology, 41*(6-7), 599-613. doi:10.1016/j.molimm.2004.04.008

Datta, K., Franke, T. F., Chan, T. O., Makris, A., Yang, S. I., Kaplan, D. R., . . . Tsichlis, P. N. (1995). AH/PH domain-mediated interaction between Akt molecules and its potential role in Akt regulation. *Molecular and Cellular Biology, 15*(4), 2304-2310.

Deane, J. A., & Fruman, D. A. (2004). Phosphoinositide 3-kinase: diverse roles in immune cell activation. *Annual Review of Immunology, 22*, 563-598. doi:10.1146/annurev.immunol.22.012703.104721

Doza, Y. N., Cuenda, A., Thomas, G. M., Cohen, P., & Nebreda, A. R. (1995). Activation of the MAP kinase homologue RK requires the phosphorylation of Thr-180 and Tyr-182 and both residues are phosphorylated in chemically stressed KB cells. *FEBS Letters, 364*(2), 223-228.

Ehrhardt, A., David, M. D., Ehrhardt, G. R., & Schrader, J. W. (2004). Distinct mechanisms determine the patterns of differential activation of H-Ras, N-Ras, K-Ras 4B, and M-Ras by receptors for growth factors or antigen. *Molecular and Cellular Biology, 24*(14), 6311-6323. doi:10.1128/MCB.24.14.6311-6323.2004

Enslen, H., Raingeaud, J., & Davis, R. J. (1998). Selective activation of p38 mitogen-activated protein (MAP) kinase isoforms by the MAP kinase kinases MKK3 and MKK6. *Journal of Biological Chemistry, 273*(3), 1741-1748.

Flaswinkel, H., & Reth, M. (1994). Dual role of the tyrosine activation motif of the Ig-alpha protein during signal transduction via the B cell antigen receptor. *EMBO Journal, 13*(1), 83-89.

Francis, D. A., Karras, J. G., Ke, X. Y., Sen, R., & Rothstein, T. L. (1995). Induction of the Transcription Factors Nf-Kb, Ap-1 and Nf-at during B-Cell Stimulation through the Cd40 Receptor. *International Immunology, 7*(2), 151-161. doi:DOI 10.1093/intimm/7.2.151

Fu, C., Turck, C. W., Kurosaki, T., & Chan, A. C. (1998). BLNK: a central linker protein in B cell activation. *Immunity, 9*(1), 93-103.

Fuchs, S. Y., Dolan, L., Davis, R. J., & Ronai, Z. (1996). Phosphorylation-dependent targeting of c-Jun ubiquitination by Jun N-kinase. *Oncogene, 13*(7), 1531-1535.

Gallo, E. M., Cante-Barrett, K., & Crabtree, G. R. (2006). Lymphocyte calcium signaling from membrane to nucleus. *Nature Immunology, 7*(1), 25-32. doi:10.1038/ni1295

Gauld, S. B., & Cambier, J. C. (2004). Src-family kinases in B-cell development and signaling. *Oncogene, 23*(48), 8001-8006. doi:10.1038/sj.onc.1208075

Goedert, M., Cuenda, A., Craxton, M., Jakes, R., & Cohen, P. (1997). Activation of the novel stress-activated protein kinase SAPK4 by cytokines and cellular stresses is mediated by SKK3 (MKK6); Comparison of its substrate specificity with that of other SAP kinases. *EMBO Journal, 16*(12), 3563-3571. doi:DOI 10.1093/emboj/16.12.3563

Gold, M. R., Ingham, R. J., McLeod, S. J., Christian, S. L., Scheid, M. P., Duronio, V., . . . Matsuuchi, L. (2000). Targets of B-cell antigen receptor signaling: the phosphatidylinositol 3-kinase/Akt/glycogen synthase kinase-3 signaling pathway and the Rap1 GTPase. *Immunological Reviews, 176*, 47-68.

Gold, M. R., Scheid, M. P., Santos, L., Dang-Lawson, M., Roth, R. A., Matsuuchi, L., . . . Krebs, D. L. (1999). The B cell antigen receptor activates the Akt (protein kinase B)/glycogen synthase kinase-3 signaling pathway via phosphatidylinositol 3-kinase. *Journal of Immunology, 163*(4), 1894-1905.

Gratacap, M. P., Payrastre, B., Viala, C., Mauco, G., Plantavid, M., & Chap, H. (1998). Phosphatidylinositol 3,4,5-trisphosphate-dependent stimulation of phospholipase C-gamma2 is an early key event in FcgammaRIIA-mediated activation of human platelets. *Journal of Biological Chemistry, 273*(38), 24314-24321.

Gupta, N., & DeFranco, A. L. (2007). Lipid rafts and B cell signaling. *Seminars in Cell & Developmental Biology, 18*(5), 616-626. doi:10.1016/j.semcdb.2007.07.009

Gupta, N., Scharenberg, A. M., Fruman, D. A., Cantley, L. C., Kinet, J. P., & Long, E. O. (1999). The SH2 domain-containing inositol 5'-phosphatase (SHIP) recruits the p85 subunit of phosphoinositide 3-kinase during FcgammaRIIb1-mediated inhibition of B cell receptor signaling. *Journal of Biological Chemistry, 274*(11), 7489-7494.

Gupta, S., Campbell, D., Derijard, B., & Davis, R. J. (1995). Transcription factor ATF2 regulation by the JNK signal transduction pathway. *Science, 267*(5196), 389-393.

Hacker, H., & Karin, M. (2006). Regulation and function of IKK and IKK-related kinases. *Science's STKE, 2006*(357), re13. doi:10.1126/stke.3572006re13

Han, J., Luby-Phelps, K., Das, B., Shu, X., Xia, Y., Mosteller, R. D., . . . Broek, D. (1998). Role of substrates and products of PI 3-kinase in regulating activation of Rac-related guanosine triphosphatases by Vav. *Science, 279*(5350), 558-560.

Hashimoto, A., Okada, H., Jiang, A., Kurosaki, M., Greenberg, S., Clark, E. A., & Kurosaki, T. (1998). Involvement of guanosine triphosphatases and phospholipase C-gamma2 in extracellular signal-regulated kinase, c-Jun NH2-terminal kinase, and p38 mitogen-activated protein kinase activation by the B cell antigen receptor. *Journal of Experimental Medicine, 188*(7), 1287-1295.

Hatzi, K., & Melnick, A. (2014). Breaking bad in the germinal center: how deregulation of BCL6 contributes to lymphomagenesis. *Trends in Molecular Medicine, 20*(6), 343-352. doi:10.1016/j.molmed.2014.03.001

Hayden, M. S., & Ghosh, S. (2004). Signaling to NF-kappaB. *Genes & Development, 18*(18), 2195-2224. doi:10.1101/gad.1228704

Homma, Y., & Emori, Y. (1995). A dual functional signal mediator showing RhoGAP and phospholipase C-delta stimulating activities. *EMBO Journal, 14*(2), 286-291.

Huang, F., & Gu, H. (2008). Negative regulation of lymphocyte development and function by the Cbl family of proteins. *Immunological Reviews, 224*, 229-238. doi:10.1111/j.1600-065X.2008.00655.x

Ingham, R. J., Holgado-Madruga, M., Siu, C., Wong, A. J., & Gold, M. R. (1998). The Gab1 protein is a docking site for multiple proteins involved in signaling by the B cell antigen receptor. *Journal of Biological Chemistry, 273*(46), 30630-30637. doi:DOI 10.1074/jbc.273.46.30630

Ingham, R. J., Santos, L., Dang-Lawson, M., Holgado-Madruga, M., Dudek, P., Maroun, C. R., . . . Gold, M. R. (2001). The Gab1 docking protein links the b cell antigen receptor to the phosphatidylinositol 3-kinase/Akt signaling pathway and to the SHP2 tyrosine phosphatase. *Journal of Biological Chemistry, 276*(15), 12257-12265. doi:10.1074/jbc.M010590200

Ingley, E., Schneider, J. R., Payne, C. J., McCarthy, D. J., Harder, K. W., Hibbs, M. L., & Klinken, S. P. (2006). Csk-binding protein mediates sequential enzymatic down-regulation and degradation of Lyn in erythropoietin-stimulated cells. *Journal of Biological Chemistry, 281*(42), 31920-31929. doi:10.1074/jbc.M602637200

Ishiai, M., Kurosaki, M., Pappu, R., Okawa, K., Ronko, I., Fu, C., . . . Kurosaki, T. (1999). BLNK required for coupling Syk to PLC gamma 2 and Rac1-JNK in B cells. *Immunity, 10*(1), 117-125.

Iwaki, S., Jensen, B. M., & Gilfillan, A. M. (2007). Ntal/Lab/Lat2. *International Journal of Biochemistry and Cell Biology, 39*(5), 868-873. doi:10.1016/j.biocel.2006.10.018

Jiang, A., Craxton, A., Kurosaki, T., & Clark, E. A. (1998). Different protein tyrosine kinases are required for B cell antigen receptor-mediated activation of extracellular signal-regulated kinase, c-Jun NH2-terminal kinase 1, and p38 mitogen-activated protein kinase. *Journal of Experimental Medicine, 188*(7), 1297-1306.

Justement, L. B., Campbell, K. S., Chien, N. C., & Cambier, J. C. (1991). Regulation of B cell antigen receptor signal transduction and phosphorylation by CD45. *Science, 252*(5014), 1839-1842.

Justement, L. B., Wienands, J., Hombach, J., Reth, M., & Cambier, J. C. (1990). Membrane IgM and IgD molecules fail to transduce Ca2+ mobilizing signals when expressed on differentiated B lineage cells. *Journal of Immunology, 144*(9), 3272-3280.

Kang, S. W., Wahl, M. I., Chu, J., Kitaura, J., Kawakami, Y., Kato, R. M., . . . Rawlings, D. J. (2001). PKCbeta modulates antigen receptor signaling via regulation of Btk membrane localization. *EMBO Journal, 20*(20), 5692-5702. doi:10.1093/emboj/20.20.5692

Katkere, B., Rosa, S., & Drake, J. R. (2012). The Syk-binding ubiquitin ligase c-Cbl mediates signaling-dependent B cell receptor ubiquitination and B cell receptor-mediated antigen processing and presentation. *Journal of Biological Chemistry, 287*(20), 16636-16644. doi:10.1074/jbc.M112.357640

Kawabuchi, M., Satomi, Y., Takao, T., Shimonishi, Y., Nada, S., Nagai, K., . . . Okada, M. (2000). Transmembrane phosphoprotein Cbp regulates the activities of Src-family tyrosine kinases. *Nature, 404*(6781), 999-1003. doi:10.1038/35010121

Kawauchi, K., Lazarus, A. H., Sanghera, J. S., Man, G. L., Pelech, S. L., & Delovitch, T. L. (1996). Regulation of BCR- and PKC/Ca(2+)-mediated activation of the Raf1/MEK/MAPK pathway by protein-tyrosine kinase and -tyrosine phosphatase activities. *Molecular Immunology, 33*(3), 287-296.

King, C. G., Kobayashi, T., Cejas, P. J., Kim, T., Yoon, K., Kim, G. K., . . . Choi, Y. (2006). TRAF6 is a T cell-intrinsic negative regulator required for the maintenance of immune homeostasis. *Nature Medicine, 12*(9), 1088-1092. doi:10.1038/nm1449

Koncz, G., Bodor, C., Kovesdi, D., Gati, R., & Sarmay, G. (2002). BCR mediated signal transduction in immature and mature B cells. *Immunology Letters, 82*(1-2), 41-49.

Koonpaew, S., Janssen, E., Zhu, M., & Zhang, W. (2004). The importance of three membrane-distal tyrosines in the adaptor protein NTAL/LAB. *Journal of Biological Chemistry, 279*(12), 11229-11235. doi:10.1074/jbc.M311394200

Kurosaki, T., & Kurosaki, M. (1997). Transphosphorylation of Bruton's tyrosine kinase on tyrosine 551 is critical for B cell antigen receptor function. *Journal of Biological Chemistry, 272*(25), 15595-15598. doi:DOI 10.1074/jbc.272.25.15595

Kyriakis, J. M., App, H., Zhang, X. F., Banerjee, P., Brautigan, D. L., Rapp, U. R., & Avruch, J. (1992). Raf-1 activates MAP kinase-kinase. *Nature, 358*(6385), 417-421. doi:10.1038/358417a0

Law, C. L., Sidorenko, S. P., Chandran, K. A., Zhao, Z., Shen, S. H., Fischer, E. H., & Clark, E. A. (1996). CD22 associates with protein tyrosine phosphatase 1C, Syk, and phospholipase C-gamma(1) upon B cell activation. *Journal of Experimental Medicine, 183*(2), 547-560.

Leandro, M. J. (2013). B-cell subpopulations in humans and their differential susceptibility to depletion with anti-CD20 monoclonal antibodies. *Arthritis Research & Therapy, 15*. doi:ARTN S3

10.1186/ar3908

Lemay, S., Davidson, D., Latour, S., & Veillette, A. (2000). Dok-3, a novel adapter molecule involved in the negative regulation of immunoreceptor signaling. *Molecular and Cellular Biology, 20*(8), 2743-2754.

Leung, W. H., Tarasenko, T., & Bolland, S. (2009). Differential roles for the inositol phosphatase SHIP in the regulation of macrophages and lymphocytes. *Immunologic Research, 43*(1-3), 243-251. doi:10.1007/s12026-008-8078-1

Lin, A., Minden, A., Martinetto, H., Claret, F. X., Lange-Carter, C., Mercurio, F., . . . Karin, M. (1995). Identification of a dual specificity kinase that activates the Jun kinases and p38-Mpk2. *Science, 268*(5208), 286-290.

Lin, X., Cunningham, E. T., Jr., Mu, Y., Geleziunas, R., & Greene, W. C. (1999). The proto-oncogene Cot kinase participates in CD3/CD28 induction of NF-kappaB acting through the NF-kappaB-inducing kinase and IkappaB kinases. *Immunity, 10*(2), 271-280.

Liu, Q. R., Oliveira-Dos-Santos, A. J., Mariathasan, S., Bouchard, D., Jones, J., Sarao, R., . . . Dumont, D. J. (1998). The inositol polyphosphate 5-phosphatase ship is a crucial negative regulator of B cell antigen receptor signaling. *Journal of Experimental Medicine, 188*(7), 1333-1342. doi:DOI 10.1084/jem.188.7.1333

Lorenzo, P. S., Kung, J. W., Bottorff, D. A., Garfield, S. H., Stone, J. C., & Blumberg, P. M. (2001). Phorbol esters modulate the Ras exchange factor RasGRP3. *Cancer Research, 61*(3), 943-949.

Lu, X., Axtell, R. C., Collawn, J. F., Gibson, A., Justement, L. B., & Raman, C. (2002). AP2 adaptor complex-dependent internalization of CD5: differential regulation in T and B cells. *Journal of Immunology, 168*(11), 5612-5620.

Lyubchenko, T. (2010). Ca(2)+ signaling in B cells. *ScientificWorldJournal, 10*, 2254-2264. doi:10.1100/tsw.2010.219

Maeda, A., Kurosaki, M., Ono, M., Takai, T., & Kurosaki, T. (1998). Requirement of SH2-containing protein tyrosine phosphatases SHP-1 and SHP-2 for paired immunoglobulin-like receptor B (PIR-B)-mediated inhibitory signal. *Journal of Experimental Medicine, 187*(8), 1355-1360. doi:DOI 10.1084/jem.187.8.1355

Maehama, T., & Dixon, J. E. (1998). The tumor suppressor, PTEN/MMAC1, dephosphorylates the lipid second messenger, phosphatidylinositol 3,4,5-trisphosphate. *Journal of Biological Chemistry, 273*(22), 13375-13378.

Majchrzak, A., Witkowska, M., & Smolewski, P. (2014). Inhibition of the PI3K/Akt/mTOR signaling pathway in diffuse large B-cell lymphoma: current knowledge and clinical significance. *Molecules, 19*(9), 14304-14315. doi:10.3390/molecules190914304

Malhotra, S., Kovats, S., Zhang, W., & Coggeshall, K. M. (2009). Vav and Rac activation in B cell antigen receptor endocytosis involves Vav recruitment to the adapter protein LAB. *Journal of Biological Chemistry, 284*(52), 36202-36212. doi:10.1074/jbc.M109.040089

Manno, B., Oellerich, T., Schnyder, T., Corso, J., Losing, M., Neumann, K., . . . Wienands, J. (2016). The Dok-3/Grb2 adaptor module promotes inducible association of the lipid phosphatase SHIP with the BCR in a coreceptor-independent manner. *European Journal of Immunology, 46*(11), 2520-2530. doi:10.1002/eji.201646431

Marshall, A. J., Niiro, H., Lerner, C. G., Yun, T. J., Thomas, S., Disteche, C. M., & Clark, E. A. (2000). A novel B lymphocyte-associated adaptor protein, Bam32, regulates antigen receptor signaling downstream of phosphatidylinositol 3-kinase. *Journal of Experimental Medicine, 191*(8), 1319-1332.

McLeod, S. J., & Gold, M. R. (2001). Activation and function of the Rap1 GTPase in B lymphocytes. *International Reviews of Immunology, 20*(6), 763-789.

Meininger, I., & Krappmann, D. (2016). Lymphocyte signaling and activation by the CARMA1-BCL10-MALT1 signalosome. *Biological Chemistry, 397*(12), 1315-1333. doi:10.1515/hsz-2016-0216

Mitchell, S., Vargas, J., & Hoffmann, A. (2016). Signaling via the NFkappaB system. *Wiley Interdisciplinary Reviews: Systems Biology and Medicine, 8*(3), 227-241. doi:10.1002/wsbm.1331

Mizuno, K., Tagawa, Y., Mitomo, K., Arimura, Y., Hatano, N., Katagiri, T., . . . Yakura, H. (2000). Src homology region 2 (SH2) domain-containing phosphatase-1 dephosphorylates B cell linker protein/SH2 domain leukocyte protein of 65 kDa and selectively regulates c-Jun NH2-terminal kinase activation in B cells. *Journal of Immunology, 165*(3), 1344-1351.

Nagai, K., Takata, M., Yamamura, H., & Kurosaki, T. (1995). Tyrosine phosphorylation of Shc is mediated through Lyn and Syk in B cell receptor signaling. *Journal of Biological Chemistry, 270*(12), 6824-6829.

Nel, A. E., Landreth, G. E., Goldschmidtclermont, P. J., Tung, H. E., & Galbraith, R. M. (1984). Enhanced Tyrosine Phosphorylation in Lymphocytes-B Upon Complexing of Membrane Immunoglobulin. *Biochemical and Biophysical Research Communications, 125*(3), 859-866. doi:Doi 10.1016/0006-291x(84)91362-7

Niiro, H., Allam, A., Stoddart, A., Brodsky, F. M., Marshall, A. J., & Clark, E. A. (2004). The B lymphocyte adaptor molecule of 32 kilodaltons (Bam32) regulates B cell antigen receptor internalization. *Journal of Immunology, 173*(9), 5601-5609.

Niiro, H., Jabbarzadeh-Tabrizi, S., Kikushige, Y., Shima, T., Noda, K., Ota, S., . . . Akashi, K. (2012). CIN85 is required for Cbl-mediated regulation of antigen receptor signaling in human B cells. *Blood, 119*(10), 2263-2273. doi:10.1182/blood-2011-04-351965

O'Rourke, L. M., Tooze, R., Turner, M., Sandoval, D. M., Carter, R. H., Tybulewicz, V. L. J., & Fearon, D. T. (1998). CD19 as a membrane-anchored adaptor protein of B lymphocytes: Costimulation of lipid and protein kinases by recruitment of Vav. *Immunity, 8*(5), 635-645. doi:Doi 10.1016/S1074-7613(00)80568-3

Oh-hora, M., Johmura, S., Hashimoto, A., Hikida, M., & Kurosaki, T. (2003). Requirement for Ras guanine nucleotide releasing protein 3 in coupling phospholipase C-gamma2 to Ras in B cell receptor signaling. *Journal of Experimental Medicine, 198*(12), 1841-1851. doi:10.1084/jem.20031547

Okada, T., Maeda, A., Iwamatsu, A., Gotoh, K., & Kurosaki, T. (2000). BCAP: The tyrosine kinase substrate that connects B cell receptor to phosphoinositide 3-kinase activation. *Immunity, 13*(6), 817-827. doi:Doi 10.1016/S1074-7613(00)00079-0

Otipoby, K. L., Draves, K. E., & Clark, E. A. (2001). CD22 regulates B cell receptor-mediated signals via two domains that independently recruit Grb2 and SHP-1. *Journal of Biological Chemistry, 276*(47), 44315-44322. doi:10.1074/jbc.M105446200

Pani, G., Kozlowski, M., Cambier, J. C., Mills, G. B., & Siminovitch, K. A. (1995). Identification of the Tyrosine Phosphatase Ptp1c as a B-Cell Antigen Receptor-Associated Protein Involved in the Regulation of B-Cell Signaling. *Journal of Experimental Medicine, 181*(6), 2077-2084. doi:DOI 10.1084/jem.181.6.2077

Pao, L. I., Bedzyk, W. D., Persin, C., & Cambier, J. C. (1997a). Molecular targets of CD45 in B cell antigen receptor signal transduction. *Journal of Immunology, 158*(3), 1116-1124.

Pao, L. I., Bedzyk, W. D., Persin, C., & Cambier, J. C. (1997b). Molecular targets of CD45 in B cell antigen receptor signal transduction. *Journal of Immunology, 158*(3), 1116-1124.

Pao, L. I., & Cambier, J. C. (1997). Syk, but not Lyn, recruitment to B cell antigen receptor and activation following stimulation of CD45(-) B cells. *Journal of Immunology, 158*(6), 2663-2669.

Pao, L. I., Famiglietti, S. J., & Cambier, J. C. (1998). Asymmetrical phosphorylation and function of immunoreceptor tyrosine-based activation motif tyrosines in B cell antigen receptor signal transduction. *Journal of Immunology, 160*(7), 3305-3314.

Paul, S., & Schaefer, B. C. (2013). A new look at T cell receptor signaling to nuclear factor-kappaB. *Trends in Immunology, 34*(6), 269-281. doi:10.1016/j.it.2013.02.002

Pleiman, C. M., Abrams, C., Gauen, L. T., Bedzyk, W., Jongstra, J., Shaw, A. S., & Cambier, J. C. (1994). Distinct p53/56lyn and p59fyn domains associate with nonphosphorylated and phosphorylated Ig-alpha. *Proceedings of the National Academy of Sciences of the United States of America, 91*(10), 4268-4272.

Pore, D., & Gupta, N. (2015). The ezrin-radixin-moesin family of proteins in the regulation of B-cell immune response. *Critical Reviews in Immunology, 35*(1), 15-31.

Ravichandran, K. S., Lorenz, U., Shoelson, S. E., & Burakoff, S. J. (1995). Interaction of Shc with Grb2 regulates association of Grb2 with mSOS. *Molecular and Cellular Biology, 15*(2), 593-600.

Rawlings, D. J., Scharenberg, A. M., Park, H., Wahl, M. I., Lin, S., Kato, R. M., . . . Kinet, J. P. (1996). Activation of BTK by a phosphorylation mechanism initiated by SRC family kinases. *Science, 271*(5250), 822-825.

Richards, J. D., Dave, S. H., Chou, C. H., Mamchak, A. A., & DeFranco, A. L. (2001). Inhibition of the MEK/ERK signaling pathway blocks a subset of B cell responses to antigen. *Journal of Immunology, 166*(6), 3855-3864.

Sabapathy, K., Hochedlinger, K., Nam, S. Y., Bauer, A., Karin, M., & Wagner, E. F. (2004). Distinct roles for JNK1 and JNK2 in regulating JNK activity and c-Jun-dependent cell proliferation. *Molecular Cell, 15*(5), 713-725. doi:10.1016/j.molcel.2004.08.028

Saito, K., Scharenberg, A. M., & Kinet, J. P. (2001). Interaction between the Btk PH domain and phosphatidylinositol-3,4,5-trisphosphate directly regulates Btk. *Journal of Biological Chemistry, 276*(19), 16201-16206. doi:DOI 10.1074/jbc.M100873200

Saito, K., Tolias, K. F., Saci, A., Koon, H. B., Humphries, L. A., Scharenberg, A., . . . Carpenter, C. L. (2003). BTK regulates PtdIns-4,5-P2 synthesis: importance for calcium signaling and PI3K activity. *Immunity, 19*(5), 669-678.

Sato, S., Miller, A. S., Howard, M. C., & Tedder, T. F. (1997). Regulation of B lymphocyte development and activation by the CD19/CD21/CD81/Leu 13 complex requires the cytoplasmic domain of CD19. *Journal of Immunology, 159*(7), 3278-3287.

Saunders, A. E., & Johnson, P. (2010). Modulation of immune cell signalling by the leukocyte common tyrosine phosphatase, CD45. *Cellular Signalling, 22*(3), 339-348. doi:10.1016/j.cellsig.2009.10.003

Shinohara, H., Yasuda, T., Aiba, Y., Sanjo, H., Hamadate, M., Watarai, H., . . . Kurosaki, T. (2005). PKC beta regulates BCR-mediated IKK activation by facilitating the interaction between TAK1 and CARMA1. *Journal of Experimental Medicine, 202*(10), 1423-1431. doi:DOI 10.1084/jem.20051591

Smith, K. G. C., Tarlinton, D. M., Doody, G. M., Hibbs, M. L., & Fearon, D. T. (1998). Inhibition of the B cell by CD22: A requirement for Lyn. *Journal of Experimental Medicine, 187*(5), 807-811. doi:DOI 10.1084/jem.187.5.807

Sohn, H. W., Gu, H., & Pierce, S. K. (2003). Cbl-b negatively regulates B cell antigen receptor signaling in mature B cells through ubiquitination of the tyrosine kinase Syk. *Journal of Experimental Medicine, 197*(11), 1511-1524. doi:10.1084/jem.20021686

Song, W., Liu, C., Seeley-Fallen, M. K., Miller, H., Ketchum, C., & Upadhyaya, A. (2013). Actin-mediated feedback loops in B-cell receptor signaling. *Immunological Reviews, 256*(1), 177-189. doi:10.1111/imr.12113

Stephens, L. R., Anderson, K. E., & Hawkins, P. T. (2001). Src family kinases mediate receptor-stimulated, phosphoinositide 3-kinase-dependent, tyrosine phosphorylation of dual adaptor for phosphotyrosine and 3-phosphoinositides-1 in endothelial and B cell lines. *Journal of Biological Chemistry, 276*(46), 42767-42773. doi:10.1074/jbc.M107194200

Stoddart, A., Dykstra, M. L., Brown, B. K., Song, W., Pierce, S. K., & Brodsky, F. M. (2002). Lipid rafts unite signaling cascades with clathrin to regulate BCR internalization. *Immunity, 17*(4), 451-462.

Stone, J. C. (2011). Regulation and Function of the RasGRP Family of Ras Activators in Blood Cells. *Genes & Cancer, 2*(3), 320-334. doi:10.1177/1947601911408082

Stork, B., Neumann, K., Goldbeck, I., Alers, S., Kahne, T., Naumann, M., . . . Wienands, J. (2007). Subcellular localization of Grb2 by the adaptor protein Dok-3 restricts the intensity of Ca2+ signaling in B cells. *EMBO Journal, 26*(4), 1140-1149. doi:10.1038/sj.emboj.7601557

Sutherland, C. L., Heath, A. W., Pelech, S. L., Young, P. R., & Gold, M. R. (1996). Differential activation of the ERK, JNK, and p38 mitogen-activated protein kinases by CD40 and the B cell antigen receptor. *Journal of Immunology, 157*(8), 3381-3390.

Swart, J. M., Bergeron, D. M., & Chiles, T. C. (2000). Identification of a membrane Ig-induced p38 mitogen-activated protein kinase module that regulates cAMP response element binding protein phosphorylation and transcriptional activation in CH31 B cell lymphomas. *Journal of Immunology, 164*(5), 2311-2319.

Szydlowski, M., Jablonska, E., & Juszczynski, P. (2014). FOXO1 Transcription Factor: A Critical Effector of the PI3K-AKT Axis in B-Cell Development. *International Reviews of Immunology, 33*(2), 146-157. doi:10.3109/08830185.2014.885022

Tamir, I., Stolpa, J. C., Helgason, C. D., Nakamura, K., Bruhns, P., Daeron, M., & Cambier, J. C. (2000). The RasGAP-binding protein p62dok is a mediator of inhibitory FcgammaRIIB signals in B cells. *Immunity, 12*(3), 347-358.

Tedder, T. F., Inaoki, M., & Sato, S. (1997). The CD19-CD21 complex regulates signal transduction thresholds governing humoral immunity and autoimmunity. *Immunity, 6*(2), 107-118. doi:Doi 10.1016/S1074-7613(00)80418-5

Tedder, T. F., Poe, J. C., & Haas, K. M. (2005). CD22: A multifunctional receptor that regulates B lymphocyte survival and signal transduction. *Advances in Immunology, Vol 88, 88*, 1-50. doi:10.1016/S0065-2776(05)88001-0

Thome, M. (2004). CARMA1, BCL-10 and MALT1 in lymphocyte development and activation. *Nature Reviews: Immunology, 4*(5), 348-359. doi:10.1038/nri1352

Traub, L. M. (2003). Sorting it out: AP-2 and alternate clathrin adaptors in endocytic cargo selection. *Journal of Cell Biology, 163*(2), 203-208. doi:10.1083/jcb.200309175

Tse, K. W., Dang-Lawson, M., Lee, R. L., Vong, D., Bulic, A., Buckbinder, L., & Gold, M. R. (2009). B cell receptor-induced phosphorylation of Pyk2 and focal adhesion kinase involves integrins and the Rap GTPases and is required for B cell spreading. *Journal of Biological Chemistry, 284*(34), 22865-22877. doi:10.1074/jbc.M109.013169

Turner, M., & Billadeau, D. D. (2002). VAV proteins as signal integrators for multi-subunit immune-recognition receptors. *Nature Reviews: Immunology, 2*(7), 476-486. doi:10.1038/nri840

Valentine, M. A., Czernik, A. J., Rachie, N., Hidaka, H., Fisher, C. L., Cambier, J. C., & Bomsztyk, K. (1995). Anti-immunoglobulin M activates nuclear calcium/calmodulin-dependent protein kinase II in human B lymphocytes. *Journal of Experimental Medicine, 182*(6), 1943-1949.

van Noesel, C. J., Lankester, A. C., van Schijndel, G. M., & van Lier, R. A. (1993). The CR2/CD19 complex on human B cells contains the src-family kinase Lyn. *International Immunology, 5*(7), 699-705.

Vega, F., Medeiros, L. J., Leventaki, V., Atwell, C., Cho-Vega, J. H., Tian, L., . . . Rassidakis, G. Z. (2006). Activation of mammalian target of rapamycin signaling pathway contributes to tumor cell survival in anaplastic lymphoma kinase-positive anaplastic large cell lymphoma. *Cancer Research, 66*(13), 6589-6597. doi:10.1158/0008-5472.CAN-05-3018

Wang, C., Deng, L., Hong, M., Akkaraju, G. R., Inoue, J., & Chen, Z. J. (2001). TAK1 is a ubiquitin-dependent kinase of MKK and IKK. *Nature, 412*(6844), 346-351. doi:10.1038/35085597

Wang, J. C., Lee, J. Y., Christian, S., Dang-Lawson, M., Pritchard, C., Freeman, S. A., & Gold, M. R. (2017). The Rap1-cofilin-1 pathway coordinates actin reorganization and MTOC polarization at the B cell immune synapse. *Journal of Cell Science, 130*(6), 1094-1109. doi:10.1242/jcs.191858

Weiss, C., Schneider, S., Wagner, E. F., Zhang, X., Seto, E., & Bohmann, D. (2003). JNK phosphorylation relieves HDAC3-dependent suppression of the transcriptional activity of c-Jun. *EMBO Journal, 22*(14), 3686-3695. doi:10.1093/emboj/cdg364

Wu, C. J., & Ashwell, J. D. (2008). NEMO recognition of ubiquitinated Bcl10 is required for T cell receptor-mediated NF-kappaB activation. *Proceedings of the National Academy of Sciences of the United States of America, 105*(8), 3023-3028. doi:10.1073/pnas.0712313105

Wu, H. J., & Bondada, S. (2009). CD72, a Coreceptor with Both Positive and Negative Effects on B Lymphocyte Development and Function. *Journal of Clinical Immunology, 29*(1), 12-21. doi:10.1007/s10875-008-9264-6

Yasuda, T. (2016). MAP Kinase Cascades in Antigen Receptor Signaling and Physiology. *Current Topics in Microbiology and Immunology, 393*, 211-231. doi:10.1007/82_2015_481

Yasuda, T., Tezuka, T., Maeda, A., Inazu, T., Yamanashi, Y., Gu, H., . . . Yamamoto, T. (2002). Cbl-b positively regulates Btk-mediated activation of phospholipase C-gamma2 in B cells. *Journal of Experimental Medicine, 196*(1), 51-63.

Yusuf, I., Zhu, X., Kharas, M. G., Chen, J., & Fruman, D. A. (2004). Optimal B-cell proliferation requires phosphoinositide 3-kinase-dependent inactivation of FOXO transcription factors. *Blood, 104*(3), 784-787. doi:10.1182/blood-2003-09-3071

Zhao, M., New, L., Kravchenko, V. V., Kato, Y., Gram, H., di Padova, F., . . . Han, J. H. (1999). Regulation of the MEF2 family of transcription factors by p38. *Molecular and Cellular Biology, 19*(1), 21-30.

Zheng, Y., Liu, H., Coughlin, J., Zheng, J., Li, L., & Stone, J. C. (2005). Phosphorylation of RasGRP3 on threonine 133 provides a mechanistic link between PKC and Ras signaling systems in B cells. *Blood, 105*(9), 3648-3654. doi:10.1182/blood-2004-10-3916
