# Supplementary material for: Simulation of the Dynamics of Primary Immunodeficiencies in B Cells
Source: Front Immunol. 2018 Aug 2;9:1785. doi: 10.3389/fimmu.2018.01785 (PMC6082931; doi:10.3389/fimmu.2018.01785)
Supplement: Table S2 — Signaling loops from the B cell interaction network of the Boolean network. The table consists of the number signaling loops that each node was a part of. All the loops included primary immunodeficiency proteins. All input nodes were excluded from the table. Signal1 (antigen) and CD40LG (Signal 2) were the source of all loops, and the rest of the input signals did not occur in any loop. [file Table_2.docx]

# Table of activated targets by interacting effectors

| **Targets** | **Factors** | **References** |
| --- | --- | --- |
| ACTIN | ARPC2 && ARPC3 | (J. C. Wang et al., 2017) |
| AKT1 | PIP3 && PIP2_2 && PDPK1 | (Aman, Lamkin, Okada, Kurosaki, & Ravichandran, 1998; Datta et al., 1995) |
| AP2M1 | INPUT SIGNAL | (Lu et al., 2002) |
| ARPC2 | WASP | (Song et al., 2013) |
| ARPC3 | WASP | (Song et al., 2013) |
| ATF2 | MAPK8 | (S. Gupta, Campbell, Derijard, & Davis, 1995) |
| BCL10 | TRAF6 && CARD11 | (King et al., 2006; Paul & Schaefer, 2013; Thome, 2004) |
| BCR | SIGNAL1 && LYN && ~PTPN6 && ~PTPN11 && ~FCGR2B | (Daeron, 1997; Flaswinkel & Reth, 1994; Gauld & Cambier, 2004; Liu et al., 1998; Nel, Landreth, Goldschmidtclermont, Tung, & Galbraith, 1984; Pani, Kozlowski, Cambier, Mills, & Siminovitch, 1995; Szydlowski, Jablonska, & Juszczynski, 2014) |
| BLNK | SYK | (Fu, Turck, Kurosaki, & Chan, 1998; Ishiai et al., 1999; Mizuno et al., 2000) |
| BTK | BLNK && SYK && LYN && PIP3 && ~PTPN11 | (Baba et al., 2001; Buhl & Cambier, 1999; Kang et al., 2001; Kurosaki & Kurosaki, 1997; Rawlings et al., 1996; Saito et al., 2001) (Baba et al., 2001; Buhl & Cambier, 1999; Kang et al., 2001; Kurosaki & Kurosaki, 1997; Rawlings et al., 1996; Saito, Scharenberg, & Kinet, 2001) |
| CA | IP3 && IP3R && STIM1 && ORAI1 && CRACR2A && MS4A1 | (Lyubchenko, 2010) |
| CALM1 | CA | (Gallo, Cante-Barrett, & Crabtree, 2006) |
| CAMK | CALM1 && CA | (Gallo et al., 2006) |
| CaN | CALM1 && CA | (Gallo et al., 2006) |
| CARD11 | PRKCB | (Shinohara et al., 2005) |
| CBM | CARD11 && MALT1 && BCL10 && MAP3K7 | (Meininger & Krappmann, 2016) |
| CD19 | LYN && CD21 && CD81 | (Chalupny et al., 1995; Cherukuri et al., 2004; Liu et al., 1998; van Noesel, Lankester, van Schijndel, & van Lier, 1993) |
| CD21 | INPUT SIGNAL | (Sato, Miller, Howard, & Tedder, 1997; Tedder, Inaoki, & Sato, 1997) |
| CD22 | LYN && PTPRC | (Cornall et al., 1998; Tedder, Poe, & Haas, 2005) |
| CD40 | CD40LG | (Francis, Karras, Ke, Sen, & Rothstein, 1995; Sutherland, Heath, Pelech, Young, & Gold, 1996) |
| CD40LG | INPUT SIGNAL | (Sutherland et al., 1996) |
| CD72 | INPUT SIGNAL | (H. J. Wu & Bondada, 2009) |
| CD81 | INPUT SIGNAL | (Sato et al., 1997) |
| CDC42 | VAV | (O'Rourke et al., 1998; Turner & Billadeau, 2002) |
| CFL1 | RAP1A | (J. C. Wang et al., 2017) |
| CLT | SYK && AP2M1 | (Stoddart et al., 2002; Traub, 2003) |
| CRACR2A | ORAI1 | (Lyubchenko, 2010) |
| CREB | MAPK14 | (Swart, Bergeron, & Chiles, 2000) |
| DAG | PLCG2 && PIP2_1 | (Zheng et al., 2005) |
| DAPP1 | SYK | (Anderson et al., 2000; Stephens, Anderson, & Hawkins, 2001) (Anderson et al., 2000; Stephens, Anderson, & Hawkins, 2001) |
| ELK1 | MAPK3 | (Koncz, Bodor, Kovesdi, Gati, & Sarmay, 2002) |
| ETS1 | CAMK | (Valentine et al., 1995) (Valentine et al., 1995) |
| FCGR2B | INPUT SIGNAL | (Dal Porto et al., 2004) |
| GAB1 | SYK && LAT2 | (Brdicka et al., 2002; Ingham, Holgado-Madruga, Siu, Wong, & Gold, 1998; Ingham et al., 2001) |
| GRB2 | CD22 && CD72 && SHC1 && LAT2 | (D'Ambrosio, Hippen, & Cambier, 1996; Ingham et al., 2001; Law et al., 1996; Otipoby, Draves, & Clark, 2001; Stork et al., 2007) |
| GSK3B | ~AKT1 \|\| ~PRKCB | (Astoul, Watton, & Cantrell, 1999; Christian, Sims, & Gold, 2002; Gold et al., 2000; Gold et al., 1999) |
| IBCR | CLT && ACTIN | (N. Gupta & DeFranco, 2007; Malhotra, Kovats, Zhang, & Coggeshall, 2009; Niiro et al., 2004; Stoddart et al., 2002) |
| IKK | IKKA && NEMO && MAP3K7 && IKKB | (Hacker & Karin, 2006; X. Lin, Cunningham, Mu, Geleziunas, & Greene, 1999; Meininger & Krappmann, 2016) |
| IKKA | INPUT SIGNAL | (Hacker & Karin, 2006) |
| IKKB | MAP3K7 | (Meininger & Krappmann, 2016; C. Wang et al., 2001) |
| INPP5D | INPUT SIGNAL | (Aman et al., 1998) |
| IP3 | PLCG2 && PIP2_1 | (Justement, Campbell, Chien, & Cambier, 1991; Justement, Wienands, Hombach, Reth, & Cambier, 1990; Saunders & Johnson, 2010) |
| IP3R | IP3 | (Lyubchenko, 2010) |
| JUN | MAPK8 | (Fuchs, Dolan, Davis, & Ronai, 1996; Weiss et al., 2003) |
| KRAS | SOS \|\| RASGRP3 \|\| PRKCB \|\| ~RASA1 | (Caloca, Zugaza, Matallanas, Crespo, & Bustelo, 2003; Y. Chen et al., 2016; Chiu, Dalton, Ishiai, Kurosaki, & Chan, 2002; Ehrhardt, David, Ehrhardt, & Schrader, 2004; Ishiai et al., 1999; Jiang, Craxton, Kurosaki, & Clark, 1998; Oh-hora, Johmura, Hashimoto, Hikida, & Kurosaki, 2003; Tamir et al., 2000) |
| LAT2 | GRB2 \|\| SYK | (Arana et al., 2008; Brdicka et al., 2002; Iwaki, Jensen, & Gilfillan, 2007; Koonpaew, Janssen, Zhu, & Zhang, 2004) |
| LYN | SIGNAL1 && PTPRC && MS4A1 && ~PTPN6 | (Dal Porto et al., 2004; Ingley et al., 2006; Pao & Cambier, 1997; Pleiman et al., 1994) |
| MALT1 | TRAF2 | (Z. J. Chen, 2012; King et al., 2006; Paul & Schaefer, 2013; Thome, 2004) |
| MAP2K1 | RAF1 && CD19 | (Kawauchi et al., 1996; Kyriakis et al., 1992) |
| MAP2K6 | MAP3K1 | (Yasuda, 2016) |
| MAP3K1 | RAC1 \|\| CDC42 \|\| TRAF2 | (Han et al., 1998; Yasuda, 2016) |
| MAP3K7 | TAB1 && TAB2 && CARD11 && TRAF2 | (Shinohara et al., 2005; C. Wang et al., 2001) |
| MAPK14 | (MAP2K6 \|\| MAP2K4) && PRKCB | (Cuenda, Cohen, BueeScherrer, & Goedert, 1997; Doza, Cuenda, Thomas, Cohen, & Nebreda, 1995; Enslen, Raingeaud, & Davis, 1998; Goedert, Cuenda, Craxton, Jakes, & Cohen, 1997; A. Lin et al., 1995) |
| MAPK3 | MAP2K1 \|\| MAP2K4 | (Sutherland et al., 1996) |
| MAPK8 | MAP2K6 && MAP2K4 | (Yasuda, 2016) |
| MEF2C | MAPK14 && CA | (Blaeser, Ho, Prywes, & Chatila, 2000; Zhao et al., 1999) |
| MS4A1 | INPUT SIGNAL | (Leandro, 2013) |
| MTOR | AKT1 | (Majchrzak, Witkowska, & Smolewski, 2014) |
| NEMO | CBM && BCL10 && MALT1 && TRAF6 | (Hayden & Ghosh, 2004; Meininger & Krappmann, 2016; C. J. Wu & Ashwell, 2008) |
| NFAT | CaN && ~GSK3B | (Beurel, Michalek, & Jope, 2010; Gallo et al., 2006) |
| NFKB1 | ~NFKBIA | (Hayden & Ghosh, 2004) |
| NFKBIA | ~IKK | (Hayden & Ghosh, 2004) |
| ORAI1 | STIM1 | (Lyubchenko, 2010) |
| PDPK1 | PIP2_2 && PIP3 | (Gratacap et al., 1998; Saito et al., 2001) |
| PI3K | GAB1 \|\| PIK3AP1 \|\| CD19 | (Buhl & Cambier, 1999; Ingham et al., 2001; O'Rourke et al., 1998; Okada, Maeda, Iwamatsu, Gotoh, & Kurosaki, 2000) |
| PIK3AP1 | LYN && SYK | (Okada et al., 2000) |
| PIP2_1 | INPUT SIGNAL | (Saito et al., 2003) |
| PIP3 | PI3K && PIP2_1 && ~PTEN | (Deane & Fruman, 2004; N. Gupta et al., 1999; Leung, Tarasenko, & Bolland, 2009; Maehama & Dixon, 1998) |
| PLCG2 | BLNK && PIP3 && DAPP1 && SYK && BTK | (Bae et al., 1998; Chiu et al., 2002; Gratacap et al., 1998; Ishiai et al., 1999; Marshall et al., 2000; Yasuda et al., 2002) |
| PRKCB | DAG && CA && PDPK1 | (Kawauchi et al., 1996; Lorenzo et al., 2001; Stone, 2011) |
| PTEN | INPUT SIGNAL | (Maehama & Dixon, 1998) |
| PTPN11 | INPUT SIGNAL | (Ingham et al., 2001; Maeda, Kurosaki, Ono, Takai, & Kurosaki, 1998) |
| PTPN12 | INPUT SIGNAL | (Ingham et al., 2001; Maeda et al., 1998) |
| PTPN6 | INPUT SIGNAL | (Ingham et al., 2001; Maeda et al., 1998) |
| PTPRC | INPUT SIGNAL | (Pao, Bedzyk, Persin, & Cambier, 1997) |
| PYK2B | RAP1A | (Tse et al., 2009) |
| RAC1 | VAV && GRB2 && SOS && RAP1A | (Arana et al., 2008; D'Ambrosio et al., 1996; Hashimoto et al., 1998; Nagai, Takata, Yamamura, & Kurosaki, 1995) |
| RAF1 | KRAS | (Kawauchi et al., 1996) |
| RAP1A | DAG | (McLeod & Gold, 2001) |
| RASA1 | PRKCB | (Lorenzo et al., 2001; Tamir et al., 2000) |
| RASGRP3 | DAG && SYK | (Ehrhardt et al., 2004; Lorenzo et al., 2001; Zheng et al., 2005) |
| RPS6KB1 | MTOR && PDPK1 | (Vega et al., 2006) |
| SHC1 | SYK && LYN && ~PTPN12 | (Nagai et al., 1995; Pao, Famiglietti, & Cambier, 1998) |
| SIGNAL1 | INPUT SIGNAL | (Dal Porto et al., 2004) |
| SOS | GRB2 && LAT2 | (Brdicka et al., 2002; Chardin et al., 1993; Ravichandran, Lorenz, Shoelson, & Burakoff, 1995) |
| STIM1 | IP3R | (Lyubchenko, 2010) |
| SYK | BCR && LYN && ~PTPN11 | (Huang & Gu, 2008; Katkere, Rosa, & Drake, 2012; Niiro et al., 2012; Pao et al., 1997; Pao & Cambier, 1997; Sohn, Gu, & Pierce, 2003) |
| TAB1 | TRAF6 | (Hacker & Karin, 2006; X. Lin et al., 1999; Meininger & Krappmann, 2016) |
| TAB2 | TRAF6 | (Hacker & Karin, 2006; X. Lin et al., 1999; Meininger & Krappmann, 2016) |
| TRAF2 | CD40 | (Hacker & Karin, 2006; X. Lin et al., 1999; Meininger & Krappmann, 2016) |
| TRAF6 | MALT1 | (Hacker & Karin, 2006; X. Lin et al., 1999; Meininger & Krappmann, 2016) |
| VAV | CD19 && GRB2 && PIP3 && LAT2 | (Caloca et al., 2003; Han et al., 1998; Malhotra et al., 2009) |
| WASP | (CDC42 \|\| RAC1) && WIPF1 | (Arana et al., 2008) |
| WIPF1 | INPUT SIGNAL | (Arana et al., 2008) |

&&, || and ~ are AND, OR and NOT gates, respectively. The targets (column 1) are activated by factors (column 2).

# References

Aman, M. J., Lamkin, T. D., Okada, H., Kurosaki, T., & Ravichandran, K. S. (1998). The inositol phosphatase SHIP inhibits Akt/PKB activation in B cells. *Journal of Biological Chemistry, 273*(51), 33922-33928.

Anderson, K. E., Lipp, P., Bootman, M., Ridley, S. H., Coadwell, J., Ronnstrand, L., . . . Hawkins, P. T. (2000). DAPP1 undergoes a PI 3-kinase-dependent cycle of plasma-membrane recruitment and endocytosis upon cell stimulation. *Current Biology, 10*(22), 1403-1412.

Arana, E., Vehlow, A., Harwood, N. E., Vigorito, E., Henderson, R., Turner, M., . . . Batista, F. D. (2008). Activation of the small GTPase Rac2 via the B cell receptor regulates B cell adhesion and immunological-synapse formation. *Immunity, 28*(1), 88-99. doi:10.1016/j.immuni.2007.12.003

Astoul, E., Watton, S., & Cantrell, D. (1999). The dynamics of protein kinase B regulation during B cell antigen receptor engagement. *Journal of Cell Biology, 145*(7), 1511-1520.

Baba, Y., Hashimoto, S., Matsushita, M., Watanabe, D., Kishimoto, T., Kurosaki, T., & Tsukada, S. (2001). BLNK mediates Syk-dependent Btk activation. *Proceedings of the National Academy of Sciences of the United States of America, 98*(5), 2582-2586. doi:10.1073/pnas.051626198

Bae, Y. S., Cantley, L. G., Chen, C. S., Kim, S. R., Kwon, K. S., & Rhee, S. G. (1998). Activation of phospholipase C-gamma by phosphatidylinositol 3,4,5-trisphosphate. *Journal of Biological Chemistry, 273*(8), 4465-4469.

Beurel, E., Michalek, S. M., & Jope, R. S. (2010). Innate and adaptive immune responses regulated by glycogen synthase kinase-3 (GSK3). *Trends in Immunology, 31*(1), 24-31. doi:10.1016/j.it.2009.09.007

Blaeser, F., Ho, N., Prywes, R., & Chatila, T. A. (2000). Ca(2+)-dependent gene expression mediated by MEF2 transcription factors. *Journal of Biological Chemistry, 275*(1), 197-209.

Brdicka, T., Imrich, M., Angelisova, P., Brdickova, N., Horvath, O., Spicka, J., . . . Horejsi, V. (2002). Non-T cell activation linker (NTAL): a transmembrane adaptor protein involved in immunoreceptor signaling. *Journal of Experimental Medicine, 196*(12), 1617-1626.

Buhl, A. M., & Cambier, J. C. (1999). Phosphorylation of CD19 Y484 and Y515, and linked activation of phosphatidylinositol 3-kinase, are required for B cell antigen receptor-mediated activation of Bruton's tyrosine kinase. *Journal of Immunology, 162*(8), 4438-4446.

Caloca, M. J., Zugaza, J. L., Matallanas, D., Crespo, P., & Bustelo, X. R. (2003). Vav mediates Ras stimulation by direct activation of the GDP/GTP exchange factor Ras GRP1. *EMBO Journal, 22*(13), 3326-3336. doi:10.1093/emboj/cdg316

Chalupny, N. J., Aruffo, A., Esselstyn, J. M., Chan, P. Y., Bajorath, J., Blake, J., . . . Tepper, M. A. (1995). Specific Binding of Fyn and Phosphatidylinositol 3-Kinase to the B-Cell Surface Glycoprotein Cd19 through Their Src Homology-2 Domains. *European Journal of Immunology, 25*(10), 2978-2984. doi:DOI 10.1002/eji.1830251040

Chardin, P., Camonis, J. H., Gale, N. W., Vanaelst, L., Schlessinger, J., Wigler, M. H., & Barsagi, D. (1993). Human Sos1 - a Guanine-Nucleotide Exchange Factor for Ras That Binds to Grb2. *Science, 260*(5112), 1338-1343. doi:DOI 10.1126/science.8493579

Chen, Y., Zheng, Y., You, X., Yu, M., Fu, G., Su, X., . . . Wang, D. (2016). Kras Is Critical for B Cell Lymphopoiesis. *Journal of Immunology, 196*(4), 1678-1685. doi:10.4049/jimmunol.1502112

Chen, Z. J. (2012). Ubiquitination in signaling to and activation of IKK. *Immunological Reviews, 246*(1), 95-106. doi:10.1111/j.1600-065X.2012.01108.x

Cherukuri, A., Shoham, T., Sohn, H. W., Levy, S., Brooks, S., Carter, R., & Pierce, S. K. (2004). The tetraspanin CD81 is necessary for partitioning of coligated CD19/CD21-B cell antigen receptor complexes into signaling-active lipid rafts. *Journal of Immunology, 172*(1), 370-380.

Chiu, C. W., Dalton, M., Ishiai, M., Kurosaki, T., & Chan, A. C. (2002). BLNK: molecular scaffolding through 'cis'-mediated organization of signaling proteins. *EMBO Journal, 21*(23), 6461-6472. doi:DOI 10.1093/emboj/cdf658

Christian, S. L., Sims, P. V., & Gold, M. R. (2002). The B cell antigen receptor regulates the transcriptional activator beta-catenin via protein kinase C-mediated inhibition of glycogen synthase kinase-3. *Journal of Immunology, 169*(2), 758-769.

Cornall, R. J., Cyster, J. G., Hibbs, M. L., Dunn, A. R., Otipoby, K. L., Clark, E. A., & Goodnow, C. C. (1998). Polygenic autoimmune traits: Lyn, CD22, and SHP-1 are limiting elements of a biochemical pathway regulating BCR signaling and selection. *Immunity, 8*(4), 497-508.

Cuenda, A., Cohen, P., BueeScherrer, V., & Goedert, M. (1997). Activation of stress-activated protein kinase-3 (SAPK3) by cytokines and cellular stresses is mediated via SAPKK3 (MKK6); Comparison of the specificities of SAPK3 and SAPK2 (RK/p38). *EMBO Journal, 16*(2), 295-305. doi:DOI 10.1093/emboj/16.2.295

D'Ambrosio, D., Hippen, K. L., & Cambier, J. C. (1996). Distinct mechanisms mediate SHC association with the activated and resting B cell antigen receptor. *European Journal of Immunology, 26*(8), 1960-1965. doi:10.1002/eji.1830260842

Daeron, M. (1997). Fc receptor biology. *Annual Review of Immunology, 15*, 203-234. doi:10.1146/annurev.immunol.15.1.203

Dal Porto, J. M., Gauld, S. B., Merrell, K. T., Mills, D., Pugh-Bernard, A. E., & Cambier, J. (2004). B cell antigen receptor signaling 101. *Molecular Immunology, 41*(6-7), 599-613. doi:10.1016/j.molimm.2004.04.008

Datta, K., Franke, T. F., Chan, T. O., Makris, A., Yang, S. I., Kaplan, D. R., . . . Tsichlis, P. N. (1995). AH/PH domain-mediated interaction between Akt molecules and its potential role in Akt regulation. *Molecular and Cellular Biology, 15*(4), 2304-2310.

Deane, J. A., & Fruman, D. A. (2004). Phosphoinositide 3-kinase: diverse roles in immune cell activation. *Annual Review of Immunology, 22*, 563-598. doi:10.1146/annurev.immunol.22.012703.104721

Doza, Y. N., Cuenda, A., Thomas, G. M., Cohen, P., & Nebreda, A. R. (1995). Activation of the MAP kinase homologue RK requires the phosphorylation of Thr-180 and Tyr-182 and both residues are phosphorylated in chemically stressed KB cells. *FEBS Letters, 364*(2), 223-228.

Ehrhardt, A., David, M. D., Ehrhardt, G. R., & Schrader, J. W. (2004). Distinct mechanisms determine the patterns of differential activation of H-Ras, N-Ras, K-Ras 4B, and M-Ras by receptors for growth factors or antigen. *Molecular and Cellular Biology, 24*(14), 6311-6323. doi:10.1128/MCB.24.14.6311-6323.2004

Enslen, H., Raingeaud, J., & Davis, R. J. (1998). Selective activation of p38 mitogen-activated protein (MAP) kinase isoforms by the MAP kinase kinases MKK3 and MKK6. *Journal of Biological Chemistry, 273*(3), 1741-1748.

Flaswinkel, H., & Reth, M. (1994). Dual role of the tyrosine activation motif of the Ig-alpha protein during signal transduction via the B cell antigen receptor. *EMBO Journal, 13*(1), 83-89.

Francis, D. A., Karras, J. G., Ke, X. Y., Sen, R., & Rothstein, T. L. (1995). Induction of the transcription factors NF-κb, Ap-1 and NFAT during B-cell stimulation through the CD40 receptor. *International Immunology, 7*(2), 151-161. doi:DOI 10.1093/intimm/7.2.151

Fu, C., Turck, C. W., Kurosaki, T., & Chan, A. C. (1998). BLNK: a central linker protein in B cell activation. *Immunity, 9*(1), 93-103.

Fuchs, S. Y., Dolan, L., Davis, R. J., & Ronai, Z. (1996). Phosphorylation-dependent targeting of c-Jun ubiquitination by Jun N-kinase. *Oncogene, 13*(7), 1531-1535.

Gallo, E. M., Cante-Barrett, K., & Crabtree, G. R. (2006). Lymphocyte calcium signaling from membrane to nucleus. *Nature Immunology, 7*(1), 25-32. doi:10.1038/ni1295

Gauld, S. B., & Cambier, J. C. (2004). Src-family kinases in B-cell development and signaling. *Oncogene, 23*(48), 8001-8006. doi:10.1038/sj.onc.1208075

Goedert, M., Cuenda, A., Craxton, M., Jakes, R., & Cohen, P. (1997). Activation of the novel stress-activated protein kinase SAPK4 by cytokines and cellular stresses is mediated by SKK3 (MKK6); Comparison of its substrate specificity with that of other SAP kinases. *EMBO Journal, 16*(12), 3563-3571. doi:DOI 10.1093/emboj/16.12.3563

Gold, M. R., Ingham, R. J., McLeod, S. J., Christian, S. L., Scheid, M. P., Duronio, V., . . . Matsuuchi, L. (2000). Targets of B-cell antigen receptor signaling: the phosphatidylinositol 3-kinase/Akt/glycogen synthase kinase-3 signaling pathway and the Rap1 GTPase. *Immunological Reviews, 176*, 47-68.

Gold, M. R., Scheid, M. P., Santos, L., Dang-Lawson, M., Roth, R. A., Matsuuchi, L., . . . Krebs, D. L. (1999). The B cell antigen receptor activates the Akt (protein kinase B)/glycogen synthase kinase-3 signaling pathway via phosphatidylinositol 3-kinase. *Journal of Immunology, 163*(4), 1894-1905.

Gratacap, M. P., Payrastre, B., Viala, C., Mauco, G., Plantavid, M., & Chap, H. (1998). Phosphatidylinositol 3,4,5-trisphosphate-dependent stimulation of phospholipase C-gamma2 is an early key event in FcgammaRIIA-mediated activation of human platelets. *Journal of Biological Chemistry, 273*(38), 24314-24321.

Gupta, N., & DeFranco, A. L. (2007). Lipid rafts and B cell signaling. *Seminars in Cell & Developmental Biology, 18*(5), 616-626. doi:10.1016/j.semcdb.2007.07.009

Gupta, N., Scharenberg, A. M., Fruman, D. A., Cantley, L. C., Kinet, J. P., & Long, E. O. (1999). The SH2 domain-containing inositol 5'-phosphatase (SHIP) recruits the p85 subunit of phosphoinositide 3-kinase during FcgammaRIIb1-mediated inhibition of B cell receptor signaling. *Journal of Biological Chemistry, 274*(11), 7489-7494.

Gupta, S., Campbell, D., Derijard, B., & Davis, R. J. (1995). Transcription factor ATF2 regulation by the JNK signal transduction pathway. *Science, 267*(5196), 389-393.

Hacker, H., & Karin, M. (2006). Regulation and function of IKK and IKK-related kinases. *Science's STKE, 2006*(357), re13. doi:10.1126/stke.3572006re13

Han, J., Luby-Phelps, K., Das, B., Shu, X., Xia, Y., Mosteller, R. D., . . . Broek, D. (1998). Role of substrates and products of PI 3-kinase in regulating activation of Rac-related guanosine triphosphatases by Vav. *Science, 279*(5350), 558-560.

Hashimoto, A., Okada, H., Jiang, A., Kurosaki, M., Greenberg, S., Clark, E. A., & Kurosaki, T. (1998). Involvement of guanosine triphosphatases and phospholipase C-gamma2 in extracellular signal-regulated kinase, c-Jun NH2-terminal kinase, and p38 mitogen-activated protein kinase activation by the B cell antigen receptor. *Journal of Experimental Medicine, 188*(7), 1287-1295.

Hayden, M. S., & Ghosh, S. (2004). Signaling to NF-kappaB. *Genes & Development, 18*(18), 2195-2224. doi:10.1101/gad.1228704

Huang, F., & Gu, H. (2008). Negative regulation of lymphocyte development and function by the Cbl family of proteins. *Immunological Reviews, 224*, 229-238. doi:10.1111/j.1600-065X.2008.00655.x

Ingham, R. J., Holgado-Madruga, M., Siu, C., Wong, A. J., & Gold, M. R. (1998). The Gab1 protein is a docking site for multiple proteins involved in signaling by the B cell antigen receptor. *Journal of Biological Chemistry, 273*(46), 30630-30637. doi:DOI 10.1074/jbc.273.46.30630

Ingham, R. J., Santos, L., Dang-Lawson, M., Holgado-Madruga, M., Dudek, P., Maroun, C. R., . . . Gold, M. R. (2001). The Gab1 docking protein links the b cell antigen receptor to the phosphatidylinositol 3-kinase/Akt signaling pathway and to the SHP2 tyrosine phosphatase. *Journal of Biological Chemistry, 276*(15), 12257-12265. doi:10.1074/jbc.M010590200

Ingley, E., Schneider, J. R., Payne, C. J., McCarthy, D. J., Harder, K. W., Hibbs, M. L., & Klinken, S. P. (2006). Csk-binding protein mediates sequential enzymatic down-regulation and degradation of Lyn in erythropoietin-stimulated cells. *Journal of Biological Chemistry, 281*(42), 31920-31929. doi:10.1074/jbc.M602637200

Ishiai, M., Kurosaki, M., Pappu, R., Okawa, K., Ronko, I., Fu, C., . . . Kurosaki, T. (1999). BLNK required for coupling Syk to PLC gamma 2 and Rac1-JNK in B cells. *Immunity, 10*(1), 117-125.

Iwaki, S., Jensen, B. M., & Gilfillan, A. M. (2007). Ntal/Lab/Lat2. *International Journal of Biochemistry and Cell Biology, 39*(5), 868-873. doi:10.1016/j.biocel.2006.10.018

Jiang, A., Craxton, A., Kurosaki, T., & Clark, E. A. (1998). Different protein tyrosine kinases are required for B cell antigen receptor-mediated activation of extracellular signal-regulated kinase, c-Jun NH2-terminal kinase 1, and p38 mitogen-activated protein kinase. *Journal of Experimental Medicine, 188*(7), 1297-1306.

Justement, L. B., Campbell, K. S., Chien, N. C., & Cambier, J. C. (1991). Regulation of B cell antigen receptor signal transduction and phosphorylation by CD45. *Science, 252*(5014), 1839-1842.

Justement, L. B., Wienands, J., Hombach, J., Reth, M., & Cambier, J. C. (1990). Membrane IgM and IgD molecules fail to transduce Ca2+ mobilizing signals when expressed on differentiated B lineage cells. *Journal of Immunology, 144*(9), 3272-3280.

Kang, S. W., Wahl, M. I., Chu, J., Kitaura, J., Kawakami, Y., Kato, R. M., . . . Rawlings, D. J. (2001). PKCbeta modulates antigen receptor signaling via regulation of Btk membrane localization. *EMBO Journal, 20*(20), 5692-5702. doi:10.1093/emboj/20.20.5692

Katkere, B., Rosa, S., & Drake, J. R. (2012). The Syk-binding ubiquitin ligase c-Cbl mediates signaling-dependent B cell receptor ubiquitination and B cell receptor-mediated antigen processing and presentation. *Journal of Biological Chemistry, 287*(20), 16636-16644. doi:10.1074/jbc.M112.357640

Kawauchi, K., Lazarus, A. H., Sanghera, J. S., Man, G. L., Pelech, S. L., & Delovitch, T. L. (1996). Regulation of BCR- and PKC/Ca(2+)-mediated activation of the Raf1/MEK/MAPK pathway by protein-tyrosine kinase and -tyrosine phosphatase activities. *Molecular Immunology, 33*(3), 287-296.

King, C. G., Kobayashi, T., Cejas, P. J., Kim, T., Yoon, K., Kim, G. K., . . . Choi, Y. (2006). TRAF6 is a T cell-intrinsic negative regulator required for the maintenance of immune homeostasis. *Nature Medicine, 12*(9), 1088-1092. doi:10.1038/nm1449

Koncz, G., Bodor, C., Kovesdi, D., Gati, R., & Sarmay, G. (2002). BCR mediated signal transduction in immature and mature B cells. *Immunology Letters, 82*(1-2), 41-49.

Koonpaew, S., Janssen, E., Zhu, M., & Zhang, W. (2004). The importance of three membrane-distal tyrosines in the adaptor protein NTAL/LAB. *Journal of Biological Chemistry, 279*(12), 11229-11235. doi:10.1074/jbc.M311394200

Kurosaki, T., & Kurosaki, M. (1997). Transphosphorylation of Bruton's tyrosine kinase on tyrosine 551 is critical for B cell antigen receptor function. *Journal of Biological Chemistry, 272*(25), 15595-15598. doi:DOI 10.1074/jbc.272.25.15595

Kyriakis, J. M., App, H., Zhang, X. F., Banerjee, P., Brautigan, D. L., Rapp, U. R., & Avruch, J. (1992). Raf-1 activates MAP kinase-kinase. *Nature, 358*(6385), 417-421. doi:10.1038/358417a0

Law, C. L., Sidorenko, S. P., Chandran, K. A., Zhao, Z., Shen, S. H., Fischer, E. H., & Clark, E. A. (1996). CD22 associates with protein tyrosine phosphatase 1C, Syk, and phospholipase C-gamma(1) upon B cell activation. *Journal of Experimental Medicine, 183*(2), 547-560.

Leandro, M. J. (2013). B-cell subpopulations in humans and their differential susceptibility to depletion with anti-CD20 monoclonal antibodies. *Arthritis Research & Therapy, 15 Suppl 1*, S3. doi:10.1186/ar3908

Leung, W. H., Tarasenko, T., & Bolland, S. (2009). Differential roles for the inositol phosphatase SHIP in the regulation of macrophages and lymphocytes. *Immunologic Research, 43*(1-3), 243-251. doi:10.1007/s12026-008-8078-1

Lin, A., Minden, A., Martinetto, H., Claret, F. X., Lange-Carter, C., Mercurio, F., . . . Karin, M. (1995). Identification of a dual specificity kinase that activates the Jun kinases and p38-Mpk2. *Science, 268*(5208), 286-290.

Lin, X., Cunningham, E. T., Jr., Mu, Y., Geleziunas, R., & Greene, W. C. (1999). The proto-oncogene Cot kinase participates in CD3/CD28 induction of NF-kappaB acting through the NF-kappaB-inducing kinase and IkappaB kinases. *Immunity, 10*(2), 271-280.

Liu, Q. R., Oliveira-Dos-Santos, A. J., Mariathasan, S., Bouchard, D., Jones, J., Sarao, R., . . . Dumont, D. J. (1998). The inositol polyphosphate 5-phosphatase ship is a crucial negative regulator of B cell antigen receptor signaling. *Journal of Experimental Medicine, 188*(7), 1333-1342. doi:DOI 10.1084/jem.188.7.1333

Lorenzo, P. S., Kung, J. W., Bottorff, D. A., Garfield, S. H., Stone, J. C., & Blumberg, P. M. (2001). Phorbol esters modulate the Ras exchange factor RasGRP3. *Cancer Research, 61*(3), 943-949.

Lu, X., Axtell, R. C., Collawn, J. F., Gibson, A., Justement, L. B., & Raman, C. (2002). AP2 adaptor complex-dependent internalization of CD5: differential regulation in T and B cells. *Journal of Immunology, 168*(11), 5612-5620.

Lyubchenko, T. (2010). Ca(2)+ signaling in B cells. *ScientificWorldJournal, 10*, 2254-2264. doi:10.1100/tsw.2010.219

Maeda, A., Kurosaki, M., Ono, M., Takai, T., & Kurosaki, T. (1998). Requirement of SH2-containing protein tyrosine phosphatases SHP-1 and SHP-2 for paired immunoglobulin-like receptor B (PIR-B)-mediated inhibitory signal. *Journal of Experimental Medicine, 187*(8), 1355-1360. doi:DOI 10.1084/jem.187.8.1355

Maehama, T., & Dixon, J. E. (1998). The tumor suppressor, PTEN/MMAC1, dephosphorylates the lipid second messenger, phosphatidylinositol 3,4,5-trisphosphate. *Journal of Biological Chemistry, 273*(22), 13375-13378.

Majchrzak, A., Witkowska, M., & Smolewski, P. (2014). Inhibition of the PI3K/Akt/mTOR signaling pathway in diffuse large B-cell lymphoma: current knowledge and clinical significance. *Molecules, 19*(9), 14304-14315. doi:10.3390/molecules190914304

Malhotra, S., Kovats, S., Zhang, W., & Coggeshall, K. M. (2009). Vav and Rac activation in B cell antigen receptor endocytosis involves Vav recruitment to the adapter protein LAB. *Journal of Biological Chemistry, 284*(52), 36202-36212. doi:10.1074/jbc.M109.040089

Marshall, A. J., Niiro, H., Lerner, C. G., Yun, T. J., Thomas, S., Disteche, C. M., & Clark, E. A. (2000). A novel B lymphocyte-associated adaptor protein, Bam32, regulates antigen receptor signaling downstream of phosphatidylinositol 3-kinase. *Journal of Experimental Medicine, 191*(8), 1319-1332.

McLeod, S. J., & Gold, M. R. (2001). Activation and function of the Rap1 GTPase in B lymphocytes. *International Reviews of Immunology, 20*(6), 763-789.

Meininger, I., & Krappmann, D. (2016). Lymphocyte signaling and activation by the CARMA1-BCL10-MALT1 signalosome. *Biological Chemistry, 397*(12), 1315-1333. doi:10.1515/hsz-2016-0216

Mizuno, K., Tagawa, Y., Mitomo, K., Arimura, Y., Hatano, N., Katagiri, T., . . . Yakura, H. (2000). Src homology region 2 (SH2) domain-containing phosphatase-1 dephosphorylates B cell linker protein/SH2 domain leukocyte protein of 65 kDa and selectively regulates c-Jun NH2-terminal kinase activation in B cells. *Journal of Immunology, 165*(3), 1344-1351.

Nagai, K., Takata, M., Yamamura, H., & Kurosaki, T. (1995). Tyrosine phosphorylation of Shc is mediated through Lyn and Syk in B cell receptor signaling. *Journal of Biological Chemistry, 270*(12), 6824-6829.

Nel, A. E., Landreth, G. E., Goldschmidtclermont, P. J., Tung, H. E., & Galbraith, R. M. (1984). Enhanced Tyrosine Phosphorylation in Lymphocytes-B Upon Complexing of Membrane Immunoglobulin. *Biochemical and Biophysical Research Communications, 125*(3), 859-866. doi:Doi 10.1016/0006-291x(84)91362-7

Niiro, H., Allam, A., Stoddart, A., Brodsky, F. M., Marshall, A. J., & Clark, E. A. (2004). The B lymphocyte adaptor molecule of 32 kilodaltons (Bam32) regulates B cell antigen receptor internalization. *Journal of Immunology, 173*(9), 5601-5609.

Niiro, H., Jabbarzadeh-Tabrizi, S., Kikushige, Y., Shima, T., Noda, K., Ota, S., . . . Akashi, K. (2012). CIN85 is required for Cbl-mediated regulation of antigen receptor signaling in human B cells. *Blood, 119*(10), 2263-2273. doi:10.1182/blood-2011-04-351965

O'Rourke, L. M., Tooze, R., Turner, M., Sandoval, D. M., Carter, R. H., Tybulewicz, V. L. J., & Fearon, D. T. (1998). CD19 as a membrane-anchored adaptor protein of B lymphocytes: Costimulation of lipid and protein kinases by recruitment of Vav. *Immunity, 8*(5), 635-645. doi:Doi 10.1016/S1074-7613(00)80568-3

Oh-hora, M., Johmura, S., Hashimoto, A., Hikida, M., & Kurosaki, T. (2003). Requirement for Ras guanine nucleotide releasing protein 3 in coupling phospholipase C-gamma2 to Ras in B cell receptor signaling. *Journal of Experimental Medicine, 198*(12), 1841-1851. doi:10.1084/jem.20031547

Okada, T., Maeda, A., Iwamatsu, A., Gotoh, K., & Kurosaki, T. (2000). BCAP: The tyrosine kinase substrate that connects B cell receptor to phosphoinositide 3-kinase activation. *Immunity, 13*(6), 817-827. doi:Doi 10.1016/S1074-7613(00)00079-0

Otipoby, K. L., Draves, K. E., & Clark, E. A. (2001). CD22 regulates B cell receptor-mediated signals via two domains that independently recruit Grb2 and SHP-1. *Journal of Biological Chemistry, 276*(47), 44315-44322. doi:10.1074/jbc.M105446200

Pani, G., Kozlowski, M., Cambier, J. C., Mills, G. B., & Siminovitch, K. A. (1995). Identification of the Tyrosine Phosphatase Ptp1c as a B-Cell Antigen Receptor-Associated Protein Involved in the Regulation of B-Cell Signaling. *Journal of Experimental Medicine, 181*(6), 2077-2084. doi:DOI 10.1084/jem.181.6.2077

Pao, L. I., Bedzyk, W. D., Persin, C., & Cambier, J. C. (1997). Molecular targets of CD45 in B cell antigen receptor signal transduction. *Journal of Immunology, 158*(3), 1116-1124.

Pao, L. I., & Cambier, J. C. (1997). Syk, but not Lyn, recruitment to B cell antigen receptor and activation following stimulation of CD45(-) B cells. *Journal of Immunology, 158*(6), 2663-2669.

Pao, L. I., Famiglietti, S. J., & Cambier, J. C. (1998). Asymmetrical phosphorylation and function of immunoreceptor tyrosine-based activation motif tyrosines in B cell antigen receptor signal transduction. *Journal of Immunology, 160*(7), 3305-3314.

Paul, S., & Schaefer, B. C. (2013). A new look at T cell receptor signaling to nuclear factor-kappaB. *Trends in Immunology, 34*(6), 269-281. doi:10.1016/j.it.2013.02.002

Pleiman, C. M., Abrams, C., Gauen, L. T., Bedzyk, W., Jongstra, J., Shaw, A. S., & Cambier, J. C. (1994). Distinct p53/56lyn and p59fyn domains associate with nonphosphorylated and phosphorylated Ig-alpha. *Proceedings of the National Academy of Sciences of the United States of America, 91*(10), 4268-4272.

Ravichandran, K. S., Lorenz, U., Shoelson, S. E., & Burakoff, S. J. (1995). Interaction of Shc with Grb2 regulates association of Grb2 with mSOS. *Molecular and Cellular Biology, 15*(2), 593-600.

Rawlings, D. J., Scharenberg, A. M., Park, H., Wahl, M. I., Lin, S., Kato, R. M., . . . Kinet, J. P. (1996). Activation of BTK by a phosphorylation mechanism initiated by SRC family kinases. *Science, 271*(5250), 822-825.

Saito, K., Scharenberg, A. M., & Kinet, J. P. (2001). Interaction between the Btk PH domain and phosphatidylinositol-3,4,5-trisphosphate directly regulates Btk. *Journal of Biological Chemistry, 276*(19), 16201-16206. doi:DOI 10.1074/jbc.M100873200

Saito, K., Tolias, K. F., Saci, A., Koon, H. B., Humphries, L. A., Scharenberg, A., . . . Carpenter, C. L. (2003). BTK regulates PtdIns-4,5-P2 synthesis: importance for calcium signaling and PI3K activity. *Immunity, 19*(5), 669-678.

Sato, S., Miller, A. S., Howard, M. C., & Tedder, T. F. (1997). Regulation of B lymphocyte development and activation by the CD19/CD21/CD81/Leu 13 complex requires the cytoplasmic domain of CD19. *Journal of Immunology, 159*(7), 3278-3287.

Saunders, A. E., & Johnson, P. (2010). Modulation of immune cell signalling by the leukocyte common tyrosine phosphatase, CD45. *Cellular Signalling, 22*(3), 339-348. doi:10.1016/j.cellsig.2009.10.003

Shinohara, H., Yasuda, T., Aiba, Y., Sanjo, H., Hamadate, M., Watarai, H., . . . Kurosaki, T. (2005). PKC beta regulates BCR-mediated IKK activation by facilitating the interaction between TAK1 and CARMA1. *Journal of Experimental Medicine, 202*(10), 1423-1431. doi:DOI 10.1084/jem.20051591

Sohn, H. W., Gu, H., & Pierce, S. K. (2003). Cbl-b negatively regulates B cell antigen receptor signaling in mature B cells through ubiquitination of the tyrosine kinase Syk. *Journal of Experimental Medicine, 197*(11), 1511-1524. doi:10.1084/jem.20021686

Song, W., Liu, C., Seeley-Fallen, M. K., Miller, H., Ketchum, C., & Upadhyaya, A. (2013). Actin-mediated feedback loops in B-cell receptor signaling. *Immunological Reviews, 256*(1), 177-189. doi:10.1111/imr.12113

Stephens, L. R., Anderson, K. E., & Hawkins, P. T. (2001). Src family kinases mediate receptor-stimulated, phosphoinositide 3-kinase-dependent, tyrosine phosphorylation of dual adaptor for phosphotyrosine and 3-phosphoinositides-1 in endothelial and B cell lines. *Journal of Biological Chemistry, 276*(46), 42767-42773. doi:10.1074/jbc.M107194200

Stoddart, A., Dykstra, M. L., Brown, B. K., Song, W., Pierce, S. K., & Brodsky, F. M. (2002). Lipid rafts unite signaling cascades with clathrin to regulate BCR internalization. *Immunity, 17*(4), 451-462.

Stone, J. C. (2011). Regulation and Function of the RasGRP Family of Ras Activators in Blood Cells. *Genes & Cancer, 2*(3), 320-334. doi:10.1177/1947601911408082

Stork, B., Neumann, K., Goldbeck, I., Alers, S., Kahne, T., Naumann, M., . . . Wienands, J. (2007). Subcellular localization of Grb2 by the adaptor protein Dok-3 restricts the intensity of Ca2+ signaling in B cells. *EMBO Journal, 26*(4), 1140-1149. doi:10.1038/sj.emboj.7601557

Sutherland, C. L., Heath, A. W., Pelech, S. L., Young, P. R., & Gold, M. R. (1996). Differential activation of the ERK, JNK, and p38 mitogen-activated protein kinases by CD40 and the B cell antigen receptor. *Journal of Immunology, 157*(8), 3381-3390.

Swart, J. M., Bergeron, D. M., & Chiles, T. C. (2000). Identification of a membrane Ig-induced p38 mitogen-activated protein kinase module that regulates cAMP response element binding protein phosphorylation and transcriptional activation in CH31 B cell lymphomas. *Journal of Immunology, 164*(5), 2311-2319.

Szydlowski, M., Jablonska, E., & Juszczynski, P. (2014). FOXO1 Transcription Factor: A Critical Effector of the PI3K-AKT Axis in B-Cell Development. *International Reviews of Immunology, 33*(2), 146-157. doi:10.3109/08830185.2014.885022

Tamir, I., Stolpa, J. C., Helgason, C. D., Nakamura, K., Bruhns, P., Daeron, M., & Cambier, J. C. (2000). The RasGAP-binding protein p62dok is a mediator of inhibitory FcgammaRIIB signals in B cells. *Immunity, 12*(3), 347-358.

Tedder, T. F., Inaoki, M., & Sato, S. (1997). The CD19-CD21 complex regulates signal transduction thresholds governing humoral immunity and autoimmunity. *Immunity, 6*(2), 107-118. doi:Doi 10.1016/S1074-7613(00)80418-5

Tedder, T. F., Poe, J. C., & Haas, K. M. (2005). CD22: A multifunctional receptor that regulates B lymphocyte survival and signal transduction. *Advances in Immunology, Vol 88, 88*, 1-50. doi:10.1016/S0065-2776(05)88001-0

Thome, M. (2004). CARMA1, BCL-10 and MALT1 in lymphocyte development and activation. *Nature Reviews: Immunology, 4*(5), 348-359. doi:10.1038/nri1352

Traub, L. M. (2003). Sorting it out: AP-2 and alternate clathrin adaptors in endocytic cargo selection. *Journal of Cell Biology, 163*(2), 203-208. doi:10.1083/jcb.200309175

Tse, K. W., Dang-Lawson, M., Lee, R. L., Vong, D., Bulic, A., Buckbinder, L., & Gold, M. R. (2009). B cell receptor-induced phosphorylation of Pyk2 and focal adhesion kinase involves integrins and the Rap GTPases and is required for B cell spreading. *Journal of Biological Chemistry, 284*(34), 22865-22877. doi:10.1074/jbc.M109.013169

Turner, M., & Billadeau, D. D. (2002). VAV proteins as signal integrators for multi-subunit immune-recognition receptors. *Nature Reviews: Immunology, 2*(7), 476-486. doi:10.1038/nri840

Valentine, M. A., Czernik, A. J., Rachie, N., Hidaka, H., Fisher, C. L., Cambier, J. C., & Bomsztyk, K. (1995). Anti-immunoglobulin M activates nuclear calcium/calmodulin-dependent protein kinase II in human B lymphocytes. *Journal of Experimental Medicine, 182*(6), 1943-1949.

van Noesel, C. J., Lankester, A. C., van Schijndel, G. M., & van Lier, R. A. (1993). The CR2/CD19 complex on human B cells contains the src-family kinase Lyn. *International Immunology, 5*(7), 699-705.

Vega, F., Medeiros, L. J., Leventaki, V., Atwell, C., Cho-Vega, J. H., Tian, L., . . . Rassidakis, G. Z. (2006). Activation of mammalian target of rapamycin signaling pathway contributes to tumor cell survival in anaplastic lymphoma kinase-positive anaplastic large cell lymphoma. *Cancer Research, 66*(13), 6589-6597. doi:10.1158/0008-5472.CAN-05-3018

Wang, C., Deng, L., Hong, M., Akkaraju, G. R., Inoue, J., & Chen, Z. J. (2001). TAK1 is a ubiquitin-dependent kinase of MKK and IKK. *Nature, 412*(6844), 346-351. doi:10.1038/35085597

Wang, J. C., Lee, J. Y., Christian, S., Dang-Lawson, M., Pritchard, C., Freeman, S. A., & Gold, M. R. (2017). The Rap1-cofilin-1 pathway coordinates actin reorganization and MTOC polarization at the B cell immune synapse. *Journal of Cell Science, 130*(6), 1094-1109. doi:10.1242/jcs.191858

Weiss, C., Schneider, S., Wagner, E. F., Zhang, X., Seto, E., & Bohmann, D. (2003). JNK phosphorylation relieves HDAC3-dependent suppression of the transcriptional activity of c-Jun. *EMBO Journal, 22*(14), 3686-3695. doi:10.1093/emboj/cdg364

Wu, C. J., & Ashwell, J. D. (2008). NEMO recognition of ubiquitinated Bcl10 is required for T cell receptor-mediated NF-kappaB activation. *Proceedings of the National Academy of Sciences of the United States of America, 105*(8), 3023-3028. doi:10.1073/pnas.0712313105

Wu, H. J., & Bondada, S. (2009). CD72, a Coreceptor with Both Positive and Negative Effects on B Lymphocyte Development and Function. *Journal of Clinical Immunology, 29*(1), 12-21. doi:10.1007/s10875-008-9264-6

Yasuda, T. (2016). MAP Kinase Cascades in Antigen Receptor Signaling and Physiology. *Current Topics in Microbiology and Immunology, 393*, 211-231. doi:10.1007/82_2015_481

Yasuda, T., Tezuka, T., Maeda, A., Inazu, T., Yamanashi, Y., Gu, H., . . . Yamamoto, T. (2002). Cbl-b positively regulates Btk-mediated activation of phospholipase C-gamma2 in B cells. *Journal of Experimental Medicine, 196*(1), 51-63.

Zhao, M., New, L., Kravchenko, V. V., Kato, Y., Gram, H., di Padova, F., . . . Han, J. H. (1999). Regulation of the MEF2 family of transcription factors by p38. *Molecular and Cellular Biology, 19*(1), 21-30.

Zheng, Y., Liu, H., Coughlin, J., Zheng, J., Li, L., & Stone, J. C. (2005). Phosphorylation of RasGRP3 on threonine 133 provides a mechanistic link between PKC and Ras signaling systems in B cells. *Blood, 105*(9), 3648-3654. doi:10.1182/blood-2004-10-3916
